# Hexaploid sweetpotato (*Ipomoea batatas* (L.) Lam.) may not be a true type to either auto- or allopolyploid

**DOI:** 10.1371/journal.pone.0229624

**Published:** 2020-03-03

**Authors:** Ming Gao, Sara Fuentes Soriano, Qinghe Cao, Xinsun Yang, Guquan Lu

**Affiliations:** 1 Cooperative Agricultural Research Center, Prairie View A&M University, Prairie View, TX, United States of America; 2 Animal and Range Sciences Department, New Mexico State University, Las Cruces, NM, United States of America; 3 Jingsu Xuzhou Sweetpotato Research Center, Xuzhou, Jiangsu, China; 4 Food Crops Institute, Hubei Academy of Agriculture Sciences, Wuhan, China; 5 Institute of Root and Tuber Crops, Zhejiang A&F University, Lin’An, Hangzhou, Zhejiang, China; Southwest University, CHINA

## Abstract

To better define the sweetpotato polyploidy, we sought to reconstruct phylogenies of its subgenomes based on hybridization networks that could trace reticulate lineages of differentiated homoeolog triplets of multiple single-copy genes. In search of such homoeolog triplets, we distinguished cDNA variants of 811 single-copy Conserved Ortholog Set II (COSII) genes from two sweetpotato clones into variation partitions specified by corresponding homologs from two *I*. *trifida* lines, *I*. *tenuissima* and *I*. *littoralis* using a phylogenetic partition method, and amplicon variants of the COSII-marker regions from 729 of these genes from two sweetpotato clones into putative homoeoallele groups using haplotype tree and the partition methods referenced by corresponding homologs from *I*. *tenuissima*. These analyses revealed partly or completely differentiated expressed-homoeologs and homoeologs from a majority of these genes with three important features. 1. Two variation types: the predominant interspecific variations (homoeoalleles), which are non-randomly clustered, differentially interspecifically conserved or sweetpotato-specific, and the minor intraspecific ones (alleles), which are randomly distributed mostly at non-interspecifically variable sites, and usually sweetpotato-specific. 2. A clear differentiation of cDNA variants of many COSII genes into the variation partition specified by *I*. *tenuissima* or *I*. *littoralis* from that by *I*. *trifida*. 3. Three species-homolog-specified and one sweetpotato-specific variation partitions among 293 different COSII cDNAs, and two or three out of the four partitions among cDNA variants of 306 COSII genes. We then constructed hybridization networks from two concatenations of 16 and 4 alignments of 8 homologous COSII cDNA regions each, which included three taxa of expressed homoeologs in a triple-partition combination from the 16 or 4 sweetpotato COSII genes and 5 taxa each of respective cDNA homologs from the three sweetpotato relatives and *I*. *nil*, and inferred a species tree embodying both networks. The species tree predicted close-relative origins of three partly differentiated sweetpotato subgenomes.

## Introduction

Sweetpotato (*Ipomoea batatas* (L.) Lam.) is the seventh largest crop by global production (FAOSTAT, 2017 data), and an important source of food, feed and industrial raw materials worldwide. A more effective genetic improvement of sweetpotato depends on advances in our understanding of its genome and genetic inheritance. Genetic and genomic researches in sweetpotato face many unique challenges such as a very large and complex hexaploid genome (2n = 6x = 90), a large number of extremely small chromosomes, uncertain polyploid origin, self- and cross-incompatibility, etc. In particular, the polyploid origin of sweetpotato has been a subject of considerable debates for the past half-century, and remains uncertain despite growing availability of genomic data, which has critically hindered our understanding of its genome organization and structure [1, 2].

Conflicting phylogenetic features in sweetpotato have led to two opposite phylogenetic inferences of its polyploid origin among various studies. Early cytological studies [3–5] demonstrated that the sweetpotato genome consisted of three closely related, but partly homologous diploid genomes, which was more consistent with an allohexaploid genome. However, Nishiyama [6, 7] proposed an “autohexaploid” sweetpotato origin from a hexaploid “*I*. *trifida*”. This inference seemed to be supported by the morphological and physiological resemblances between sweetpotato and a synthetic hexaploid generated from hybridization of a diploid (K221, B_1_B_1_genome) and a tetraploid (K222, B_2_B_2_ B_2_B_2_ genome) forms of *I*. *trifida* (H.B.K.) G. Don [8, 9]. On the other hand, morphological and taxonomic analyses of the species and hybrids in the Ipomoea Section Batatas indicated that two species, *I*. *trifida* and *I*. *triloba* L., were the closest extant relatives of sweetpotato [10], implying an allohexaploid sweetpotato of at least two closely related, but distinct evolutionary lineages. The two opposite phylogenetic inferences persisted among recent molecular phylogenetic and systematics studies. Some of the gene-tree-based analyses of phylogenetic relationships between sweetpotato and its closest relatives employing various molecular markers, such as those using ISSR and RFLP of chloroplast DNAs [11], AFLP and ITS [12], rDNA-probes in FISH [13], and ITS, SSR, and chloroplast non-coding sequences [14], supported an autohexaploid origin of sweetpotato from *I*. *trifida*. Two linkage mapping studies using low-density RAPD [15] and AFLP [16] markers also concluded an autohexaploid origin of sweetpotato based on a predominantly polysomic inheritance. Furthermore, a recent extensive phylogenetic study of chloroplast genomes and sampled single-copy nuclear regions in sweetpotato and all of its crop wild relatives seemed to strengthen that sweetpotato was an autohexaploid of a single species origin, probably from *I*. *trifida* [17]. However, other analyses of different sets of samples employing some other markers, including those using RFLP [18], RAPD [19], the single-copy β-amylase gene [20] and three types of sweetpotato *Waxy* intron 2 variants [21], seemed to favor an allohexaploid genome that were more closely related to those of not only *I*. *trifida*, but also some other relatives, *I*. *tabascana* J.A. McDonald & D.F. Austin, *I*. *littoralis* Blume and *I*. *tenuissima* Choisy.

The persistence of the two opposite phylogenetic inferences among so many types of studies at various levels of organization in sweetpotato signaled an untypical polyploidy, which may require uncommon phylogenetic analyses to resolve. Tree-based methods used in most of the previous molecular phylogenetic studies could not have accurately modeled data for true reticulate phylogeny of sweetpotato since no tree-based method can accurately model data for reticulate phylogeny [22], nor detect hybrid origin in the absence of obvious cyto-nuclear discordance [23]. Nonetheless, all the previous studies recognized very close relatedness of the three subgenomes in sweetpotato to one another, and to that of *I*. *trifida*. However, molecular and cyto-nuclear differences among the three subgenomes of sweetpotato, albeit small, do exist, and have been identified in most of the previous studies. The focal issue regarding the polyploid origin of sweetpotato, then, become whether these observed, seemingly small inter-subgenome differences were derived from variations of genetically distinct lineages within one progenitor species, probably *I*. *trifida*, which were brought together in the hexaploid genome by intraspecific crosses during speciation, or ones of closely related evolutionary lineages brought together by hybridization of close-relative species.

To further clarify the issue, we sought to reconstruct phylogenies of the diploid subgenomes in sweetpotato based on hybridization networks that could trace probable reticulate lineages of differentiated homoeolog triplets of multiple single-copy genes. In search of such homoeolog triplets, we applied a high-resolution phylogenetic partition method [24] to distinguish partial cDNA variants of a majority of 811 sweetpotato COSII (Conserved Ortholog Set II) genes into one to three out of four variation partitions by their differential interspecific conservations of statistically non-random clusters of variations with one to three of the corresponding reference homologs from two *I*. *trifida*, a *I*. *tenuissima* and a *I*. *littoralis* accessions, or by their sweetpotato-specific variations. We further distinguished homoeoallele groups among genomic amplicon variants of the COSII-marker regions from 729 of these COSII genes from two sweetpotato clones using haplotype tree and the phylogenetic partition methods referenced by corresponding homologs from *I*. *tenuissima*. We then constructed hybridization networks [22] from two concatenations of 16 and 4 alignments of 8 homologous COSII cDNA regions each, which included three taxa of expressed homoeologs in a triple-partition combination from the 16 or 4 sweetpotato COSII genes and 5 taxa each of respective cDNA homologs from the three sweetpotato relatives and *I*. *nil* (outgroup), and inferred the most parsimonious species tree that could be well fitted with both networks. These analyses demonstrated that the sweetpotato genome has three partly differentiated diploid subgenomes of close-relative origins, and could not be simplistically described as either allo-or autohexaploid.

## Materials and methods

### Plant materials

The sweetpotato SC1149-19 clone used as a hexaploid flow-cytometry reference, and for the 454-RNAseq and the Ion Torrent PGM sequencing of COSII-marker amplicons was obtained from the US-Vegetable Lab, SC. The sweetpotato ZhengHong 3 (ZH 3, PI 606266) and Jewel (PI 531122) clones used as hexaploid flow-cytometry references, *Ipomoea littoralis* Blume (PI 573335) for the flow-cytometry analysis and the 454-RNAseq, and *Ipomoea tenuissima* Choisy (PI 553012) as a diploid flow-cytometry reference, and for the 454-RNAseq and the Ion Torrent PGM sequencing of its COSII-marker amplicons were provided by the USDA-ARS Plant Genetic Resources Conservation Unit, Griffin, GA (https://npgsweb.ars-grin.gov/gringlobal/site.aspx?id=22). The sweetpotato breeding line LSP used for the Ion Torrent PGM sequencing of COSII-marker amplicons was an in-house hybrid mothered by the Jewel (PI 531122).

### Flow cytometry analyses

Flow cytometry analyses of nuclear DNA contents in three *Ipomoea* species were performed essentially following the procedure and recommendations by Doležel [25]. Briefly, suspensions of intact nuclei were isolated by chopping fresh young leaf tissues in Galbraith's buffer following the one-step protocol, stained using DAPI (4’, 6-diamidino-2-phenylindole) and digested simultaneously with RNase as recommended. The relative fluorescence of the stained nuclei was measured by using a FACSCalibur^™^ flow cytometer (BD Biosciences, CA, USA) following the protocols for measurement of relative nuclear DNA fluorescence intensity and ploidy analysis with an external standard. The stained nuclei of diploid *I*. *tenuissima* were used as an external reference standard, and its G1peak was positioned at channel 200. Three independent replica measurements were performed using tissues from different pot-grown plants. Prior to the measurements, the instrument was aligned and calibrated by following the manufacturer’s recommendations using the BD^™^ DNA QC Particles (catalog No. 349523).

### RNA extraction, transcriptome sequencing and datasets

Total RNA samples were prepared using a procedure optimized for high-starch and high-latex tissues of the tree *Ipomoea* species. Briefly, about 5 g of tissues were pulverized in liquid nitrogen, homogenized and lysed in a15 mL preheated (65°C) buffer [1M Tris-HCl (pH 7.4,10% SDS, 0.5 M EDTA, 20% PVP-40 and 0.01% β-Mercaptoethanol). After incubation at 65 ^o^C for 15 min, the suspension was mixed with 0.1 volumes of 5 M potassium acetate and 0.25 volume of cold absolute ethanol, extracted with 1 volume of cold chloroform:isoamyl alcohol by vortexing for 2–3 min, and centrifuged at 7000g for 10 min at room temperature. The supernatants were extracted with equal-volume of Phenol: Chloroform:Isoamyl-Alcohol (50:50:1) once, cold Chloroform:Isoamyl-Alcohol (50:1) twice, and then mixed with concentrated LiCl solution to a final 3 M for overnight incubation at –20°C. Raw RNAs were recovered by centrifugation at 12,000g for 20 min, washed twice with 2 ml of 2 M LiCl solution, and dissolved in 1 ml RNase-free TE buffer [10 mM Tris-HCl (pH 7.5), 2 mM EDTA]. The RNAs were recovered using routine Sodium Acetate and Ethanol precipitation, and stored in TE buffer. Three RNA pools were made by combining equimolar amounts of 42 RNA samples isolated from leaf, vine, fibrous root and storage root tissues of sweetpotato SC1149-19 plants under various growth conditions (field, pots and hydroponic) and treatments (drought, cold & viral challenges), of 8 samples from leaf, vine and root tissues of *I*. *littoralis* plants at various growth stages, and of 9 RNA samples from leaf, vine, flower and root tissues of *I*. *tenuissima* plants. Custom transcriptome sequencing libraries from the three RNA pools were prepared, partially normalized and sequenced using 800 cycle runs on a Roche 454 GS-FLX Titanium instrument by the Center for Genomics and Bioinformatics of Indiana University. The sequencing reads were cleaned of adaptor sequences, and assembled into isotigs by the center. The numbers of assembled isotigs for the RNA pools from the sweetpotato SC1149-19, *I*. *littoralis* and *I*. *tenuissima* accessions were 53,468, 29,107 and 27,666, respectively.

A collection of 2152 tomato COSSII cDNAs was obtained from the Sol Genomics Network (https://solgenomics.net) based on the COSII member list [26] (https://www.sgn.cornell.edu/markers/cosii_markers.pl). The transcriptome dataset from the sweetpotato clone Tanzania [27] was downloaded from the International Potato Center (https://research.cip.cgiar.org/confluence/display/SPGI/Home). The two datasets of predicted cDNAs from two *I*. *trifida* lines (Mx23Hm and 0431–1) [28] were downloaded from the sweetpotato GARDEN (http://sweetpotato-garden.kazusa.or.jp). The dataset of ESTs from an *Ipomoea nil* variety was from Wei, et.al [29]. All the eight cDNA datasets were converted into searchable databases in Geneious (https://www.geneious.com) [30], and used for the phylogenetic analyses.

Each of the 2,152 tomato COSII cDNAs was used to BLAST-search its homologs in the other above-mentioned seven transcriptome databases from the five *Ipomoea* species using the Blastn program (Low complexity filter, Max E-value 1e-5, Match-mismatch scoring 2–3, and Gap-cost 52) within the Geneious suit. The numbers of hits from each of the searches, even those from the two sweetpotato datasets, were usually less than 10. All the hits from the searches using one tomato query sequence were aligned by using one of four multiple alignment programs (Geneious, MUSCLE, ClustalW and MAFFT) depending on overlapping lengths and numbers of the COSII cDNA homologs, and then locally refined using the Realign Region program within the Geneious suit to yield a set of overlapping COSSII cDNA homologs from the eight datasets. In an alignment, the cDNA variants of a COSII gene from the two sweetpotato datasets were manually screened for apparent assembly chimeras by cross-referencing haplotypes (sets of sequence variations) between the COSII cDNA variants from the same sweetpotato clone, and between matching ones from the two different sweetpotato clones. A COSII cDNA variant from one sweetpotato dataset is much less likely chimeric if its haplotype could be mostly matched in one from the other independently sequenced and assembled dataset from a different sweetpotato clone. On the other hand, a sweetpotato COSII cDNA variant was regarded as an apparent chimera, and thus removed from the alignment, if the cDNA variant displayed cross-overs of parts of two or more haplotypes of other cDNA variants from one or both of the sweetpotato datasets. Additionally, paralogous cDNA variants from multiple-copy COSII genes in the initial sets of alignments were also screened. If more than six cDNA variants having two or more distinct sets of sequence variations for a COSII gene in sweetpotato, or more than two types of cDNA variants for its homologs in the diploid *I*. *tenuissima* and/or *I*. *trifida* were identified in an alignment, these cDNA variants of the COSII gene were regarded as expressed paralogs, and the alignment excluded from further analysis. For rest of the alignments, a sub-alignment block encompassing the maximal overlapping regions for all the cDNA homologs were extracted, and used for downstream partition analyses. The COSII cDNA homologs from tomato and *I*. *nil* in the alignments were used as a reference and an outgroup for the successive hybridization network analyses, respectively.

### Phylogenetic partition analyses

Stephens’s phylogenetic partition [24] is a statistical test for identifying non-random local spatial clustering of two or more variable sites carrying mutations common to only a partition of intra-or interspecific homologous DNA sequences among many others. It calculates the probability *P*(*d*≤ *d*_*o*_) for the distance (*d*) between two or more partition-associated variable sites, if originated from randomly distributed mutations, is smaller or equal to the observed distance (*d*_*o*_) between the sites in a sample (*n* nucleotides) of homologous DNA sequences. A non-random spatial clustering, when *P*(*d*≤ *d*_*o*_) at 5% or less, of two or more variable sites having mutations shared by a particular partition of interspecific homologous DNA sequences predicts a homology of these mutations (rather than homoplasy) and their flanking regions, and thus closer relatedness of the homologs within the same partition than those in a different partition.

In each of the extracted sub-alignments, two interspecific variable sites are regarded as being significantly clustered, or far apart at the 5% level if the two sites are separated by a distance *d*_*o*_ ≤ 0.025 (n—1), or *d*_*o*_ ≥ 0.776(n—1), respectively, where n should be the consensus lengths of full-length homologous cDNAs. Since the full-length cDNA homologs were unavailable for most of the alignment sets, the length of the longest partial cDNA homolog in the initial alignment was used as the “n” for the tests, which represented a much more stringent test for spatial clustering of two variable sites. For example, in an alignment block having the longest partial cDNA homolog of 1000 bp, two interspecific variable sites separated by less than 25bp, or by more than 775 bp are significantly clustered, or far apart at the 5% level, respectively. A multi-nucleotide indel is regarded as a significant spatial clustering of adjacent variable sites as it represents statistically improbable random distribution of adjacent clustered variable sites. But, an SSR site, regardless of the unit number, is treated as a single variable site. The COSII cDNA homologs from one or two *I*. *trifida* lines, I. *littoralis* and *I*. *tenuissima* in the extracted sub-alignments were first assessed for statistically non-random clusters of variations including at least one specific to the reference species at interspecific variable sites, which define three variation partitions, Itr/Itrk/, Ils/ and Itn/, respectively. The cDNA variants of about one third of the COSII genes from the two *I*. *trifida* lines displayed different species-specific mutations at the same or different interspecific variable sites, and were regarded as two sub-partitions (Itr/ or Itrk/) of the Itr/Itrk/ specific to *I*. *trifida*. The COSII cDNA variants from sweetpotato in the sub-alignments were then distinguished into the three interspecific variation partitions by their differential conservation of the statistically non-random clusters of interspecific variations in each of the reference cDNAs from the three relatives, or into an additional partition (Hp4/) by its sweetpotato-specific non-random cluster(s) of variations at the interspecific variable sites. The Circos online program (http://mkweb.bcgsc.ca/tableviewer/) was used to create the Circos graph summarizing the partition analyses.

### Amplicon sequencing of selected COSII-marker regions

The genomic amplicon sequencing of COSII-marker regions from the screened single-copy COSII genes was part of an effort for development of genotyping-by-sequencing markers in sweetpotato, which will be reported in more details elsewhere. Briefly, a total of 864 PCR degenerate or non-degenerate primer pairs in nine 96-well plates were designed by modification of the tomato COSII-marker primer pairs, and were used to amplify corresponding homologous COSII-maker regions (short introns and flanking primer-anchoring exon borders) in two sweetpotato clones (SC1149 and LSP) and the *I*. *tenuissima* accession. Based on primer-site sequences in the sweetpotato and *I*. *tenuissima* cDNA homologs of the tomato COSII cDNAs, the primer modification included corrections of non-conserved bases, uses of degenerate bases, shifting 3–10 bases away from the exon-intron junctions, and redesigning one of the primers at nearby sites to avoid too many degenerate bases in the primers for amplifications from the corresponding COSII homoeolog triplet in sweetpotato and the COSII homolog in *I*. *tenuissima*. The 864 primer pairs and their amplification efficacy were summarized in a master list provided on a data webpage (detailed below).

Amplicons from the 864 PCR amplifications each for the three genomic DNA samples were sized and quantified using the LabChip^®^ GX Touch^™^ Nucleic Acid Analyzer (PerkinElmer, San Jose, CA), and pooled in equimolar into three size fractions (60 to 250 bp, 250 to 400 bp and 400 to 1500 bp). The three amplicon pools each for the three samples were separately used to make sequencing libraries with (the 400 to 1500 bp pool) or without (the two pools of less than 400bp) further fractionation using the Ion Xpress^™^ Plus Fragment Library Kit (ThermoFisher Scientific, San Francisco, CA). Equimolar aliquots of the two non-fractionated libraries of less than 400 bp were mixed and used for templating in a single emulsion PCR using the Ion PGM^™^ Hi-Q^™^ View OT2 Kit, and their enriched beads sequenced on a single chip following the manufacturer’s 400-bp sequencing protocol. The libraries made of fractionated amplicons were size selected for ~450 to 525 bp, templated using the Ion PGM^™^ Template IA500 Kit, and their enriched beads sequenced on a single chip following the manufacturer’s 500-bp sequencing protocol. Each set of sequencing reads was quality trimmed using the sequencer’s default software, and de novo assembled into two sets of contigs separately using the CAP3 as a plugin and the Geneious assembler in the Geneious suit. All the 12 contig datasets (2 fractions x 2 assembly x 3 samples) were converted into searchable databases in the Geneious suit for Blast-searches of contigs for amplicon variants from each of the COSII genes in the two sweetpotato clones and *I*. *tenuissima*. The two independently assembled contig groups each for the amplicon variants of a COSII gene from the two sweetpotato clones were first aligned, and apparent assembly chimeras manually screened and removed by cross-referencing haplotypes between the contigs from the two assemblies and between matching ones from the two clones. The cleaned-up alignment was then aligned with the two contig groups for the homologous COSII amplicon(s) from *I*. *tenuissima* for further reduction of apparent assembly chimeras among the contigs from both species, and for variant calling and haplotype grouping. Both the haplotype tree method using the Geneious tree builder, FastTree and PHYML, and the Stephens’s phylogenetic partition were used for haplotype grouping of the aligned COSII-maker amplicon variants from sweetpotato with their homologous amplicon from *I*. *tenuissima* as a reference.

### Hybridization network construction

For hybridization network analyses, two series of alignment blocks each containing eight homologous partial cDNAs from 16 and 4 COSII genes, respectively, in five *Ipomoea* species were concatenated using the Geneious software. In the two concatenations, each of the 8 rows of 16 or 4 concatenated partial cDNAs from various COSII genes, respectively represents a taxon of sampled expressed sequences in the same variation partition from one species, and was labeled by its partitions and source species. In less than 1% of the alignment blocks, the homologous partial COSII cDNA from *I*. *nil* was shorter than the consensus length or not identified in the published dataset so that they were replaced with the homologous regions of corresponding full-length cDNAs from GenBank. Additionally, columns of “-N-” with the numbers of “N”s equal to the position number of an alignment block in the concatenations were inserted in front of the alignment block starting from the second one, which serve as identifiers of the alignment blocks, but do not interfere the network analyses. The SplitsTree4 program (http://www.splitstree.org/) was used to construct a hybridization network from each of the two concatenations. The two hybridization networks were resolved by using hybridization transformations of two split networks that were built by the split decomposition from the pairwise LogDet distances between the 8 taxa in each alignment matrix of the two concatenations. The least square fit (LSFit) measuring the degree of fit between the pairwise distances in the hybridization-network graphs and the pairwise distances in the alignment matrix were used for evaluating robustness of the hybridization networks.

## Results

### DNA ploidy of *Ipomoea littoralis*

For references used in comparative phylogenetic analyses of sweetpotato transcriptomes, we obtained deep-coverage transcriptome datasets of partial cDNAs (>800 bp in average) from *I*. *littoralis* and *I*. *tenuissima* using the long-read 454-NGS. Initial comparative analyses of COSII cDNAs from *I*. *littoralis*, *I*. *tenuissima* and *I*. *trifida* revealed two distinct lineages between cDNA variants of many COSII genes in *I*. *littoralis*, one extremely trifida or tenuissima-like and the other littoralis-specific (examples given below), which seemed to be more consistent with them being expressed from two homoeologous loci in two diploid subgenomes than from paralogous ones in a diploid genome.

**Fig 1:**
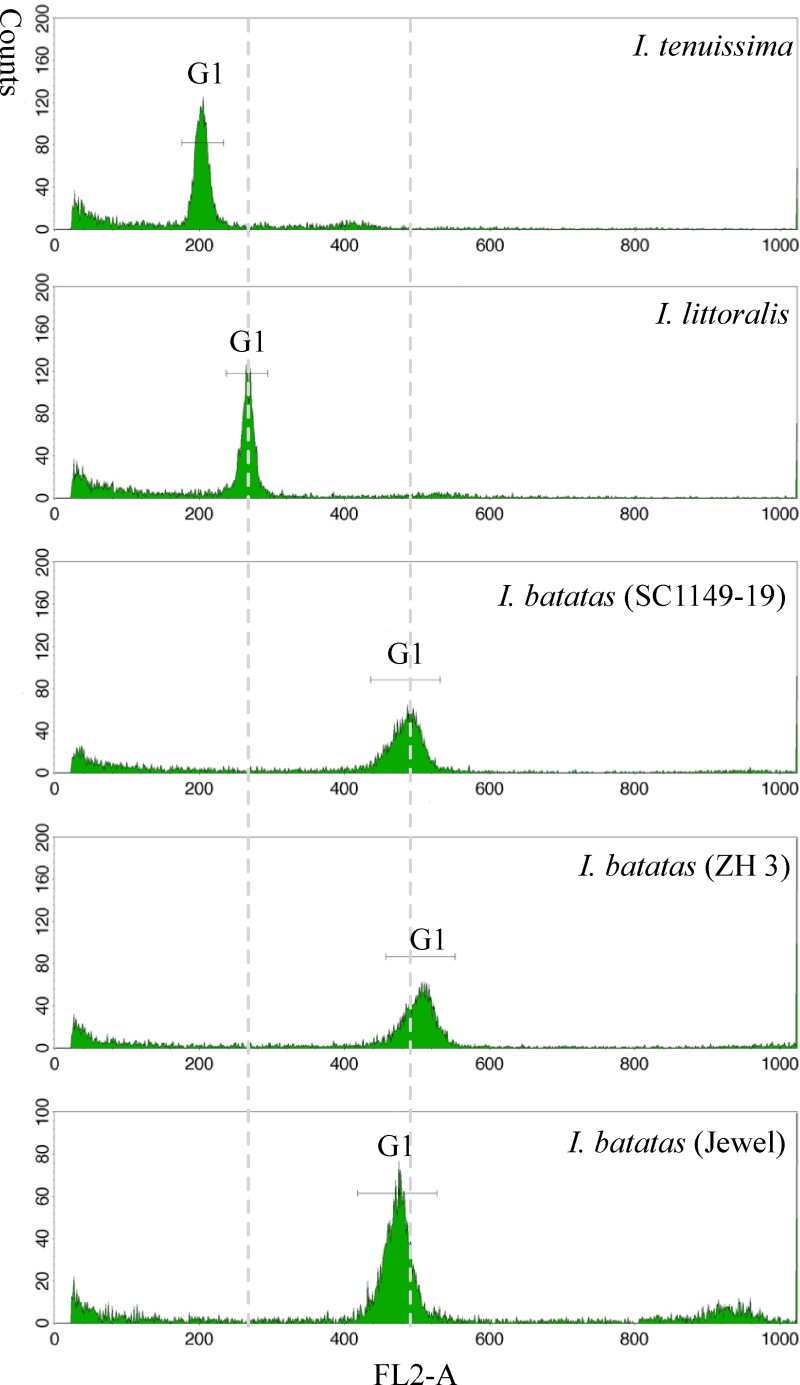
Flow-cytometry analyses of relative nuclear DNA contents in the *Ipomoea littoralis* Blume accession. Suspensions of intact nuclei were prepared from young leaf tissues, stained using DAPI and analyzed using a FACSCalibur^™^ flow cytometer. The diploid *I*. *tenuissima* was used as an external reference standard and its G1peak positioned at channel 200. The three sweetpotato clones were used as external hexaploid references.

To ascertain ploidy of the *I*. *littoralis* accession, its nuclear content relative to the diploid *I*. *tenuissima* and three hexaploid sweetpotato accessions was assessed by using flow-cytometry analyses of freshly isolated intact nuclei from young leaf tissues. Fig 1 shows the results from one of three independent analyses. The nuclear DNA content of the *I*. *littoralis* accession was an average 1.355-fold that of *I*. *tenuissima*, and average 0.506 to 0.552-fold that of the three sweetpotato clones so that it should be a 2.7x and 3 to 3.4x DNA ploidy relative to *I*. *tenuissima* and sweetpotato, respectively. Considering the presence of two distinct lineages of some COSII variants, this DNA ploidy level of the *I*. *littoralis* accession could be more reasonably explained by a massive genome reduction (or diploidization) in a tetraploid after speciation rather than by a higher nuclear DNA content in a closely related diploid, i.e. a probable paleotetraploid. The results also ruled out the suggestion that the *I*. *littoralis* accession might be a feral form of the hexaploid sweetpotato [14]. Notably, the nuclear DNA contents of the three sweetpotato accessions were average 2.29 to 2.50-fold that of *I*. *tenuissima*, equivalent to a 4.6x or 5x DNA ploidy level, which displayed a similar degree (about 1x DNA ploidy) of genome reduction after its speciation as that in *I*. *littoralis*.

### Two distinguishable types and clustered distribution of variations in cDNA variants of many sweetpotato COSII genes

**Fig 2:**
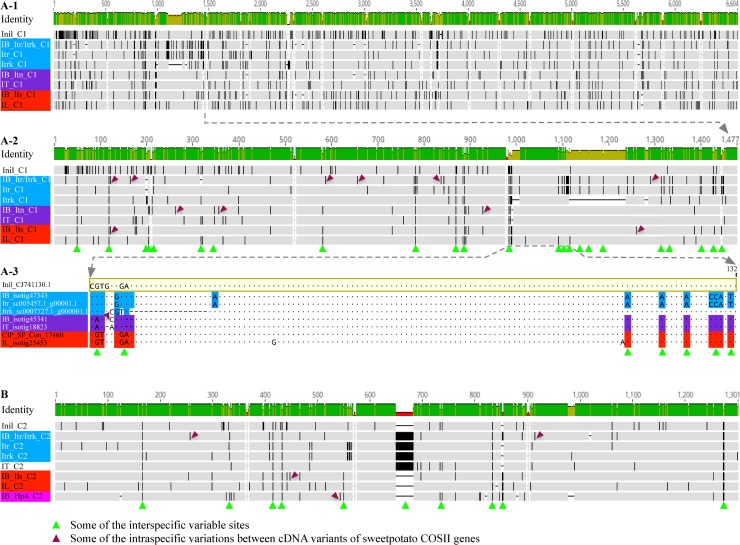
Illustration of two distinguishable forms of variations and four phylogenetic partitions among cDNA variants of some COSII genes in sweetpotato. The two alignments **A-1** and **B** in zoom-out variation-highlighting views were two concatenations (_C1 and _C2) of 16 and 4 alignment blocks, respectively, which included three cDNA variants of the 16 and 4 COSII genes from the sweetpotato SC1149-19 or Tanzania clones (combination of IB_ with_C1 or _C2), and their homologs from *I*. *tenuissima* (IT_), *I*. *trifida* (Itr_ and Itrk_ for two lines), *I*. *littoralis* (IL_) and *I*. *nil* (Inil_) in matching column-orders. Black bars highlight all sequence disagreements at various positions against the consensus in individual alignment blocks. The green and maroon triangles indicate sites of two distinguishable forms of variations in among cDNA variants of some COSII genes in sweetpotato. The three cDNA variants each of the 4 and 16 sweetpotato COSII genes carried differential non-random clusters of variations that are either well conserved in one of the reference homologs from *I*. *trifida*, *I*. *tenuissima* and *I*. *littoralis* (as in **A**), or unshared (_Hp4_ in **B**) at the common interspecific variable sites, and could be thus distinguished into four species-specified phylogenetic partitions, which were marked with _Itr/Itrk_, _Itn_, _Ils_ and _Hp4_, and shaded with blue, purple, red and pink colors, respectively. The **A-2** and **A-3** alignments are zoom-in views of the first four concatenated blocks, and the first 132-bp (consensus length) of the fourth block in the first concatenation, respectively. The **A-3** alignment also illustrates detailed phylogenetic variation partitions of three cDNA variants of the sweetpotato COSII gene (IB_ and CIP_ for isotigs from the SC1149-19 and Tanzania clones, respectively) with corresponding cDNA homologs from the three relatives.

We first sought to assess phylogenetic differentiation among homoeolog triplets of screened single-copy COSII genes in sweetpotato through detailed comparisons of variations among their cDNA variants with those in respective homologs from three of sweetpotato’s closest relatives. For the analyses, we obtained 811 alignments of equal-consensus-length homologous regions from partial cDNA variants of 811 single-copy COSII genes in two sweetpotato clones, and from their corresponding cDNA homologs from two *I*. *trifida*, *I*. *littoralis* and *I*. *tenuissima*, *I*. *nil* and tomato lines, by screening alignments of overlapping homologous cDNAs from 2152 COSII genes in these species and lines. The rest of 1341 alignments were not selected due to presence of apparent expressed paralogs or too many apparent assembly chimeras of sweetpotato COSII genes, or lack of one or more partition reference cDNAs. In the 811 alignments, the pair-wise sequence variations between COSII cDNA variants from sweetpotato, and between COSII cDNA homologs from sweetpotato and its three closest relatives, were mostly less than 3% and 5%, respectively. However, variations between cDNA variants of many sweetpotato COSII genes, albeit small, displayed two important patterns, as illustrated in the variation-highlighting views of two concatenations of homologous cDNA regions from 16 and 4 COSII genes, respectively, in the five *Ipomoea* species (Fig 2). The first concatenation (variation highlighting view in Fig 2A, and sequence in Supplemental S2 Fig) comprised three taxa (IB_Itr/Itrk_C1, IB_Itn_C1 and IB_Ils_C1) of partial cDNA variants of 16 sweetpotato COSSII genes in the Itr/Itrk/, Itn/ and Ils/ partitions (detailed below), and five taxa of corresponding partial COSII cDNA homologs from the two *I*. *trifida* lines (Itr_C1 and Itrk_C1), *I*. *tenuissima* (It_C1), *I*. *littoralis* (IL-C1), and *I*. *nil* (Inil_C1, outgroup) lines. The second concatenation (variation highlighting view in Fig 2B, and sequence in Supplemental S3 Fig) comprised three taxa (IB_Itr/Itrk_C2, IB_Hp4_C2 and IB_Ils_C2) of partial cDNA variants of 4 sweetpotato COSSII genes in the Itr/Itrk/, Hp4/ and Ils/ partitions (detailed below), and five taxa of corresponding partial cDNA homologs from the two *I*. *trifida* lines (Itr_C2 and Itrk_C2), *I*. *tenuissima* (IT_C2) *I*. *littoralis* (IL-C2) and *I*. *nil* (Inil_C2, as an outgroup) lines.

**Fig 3:**
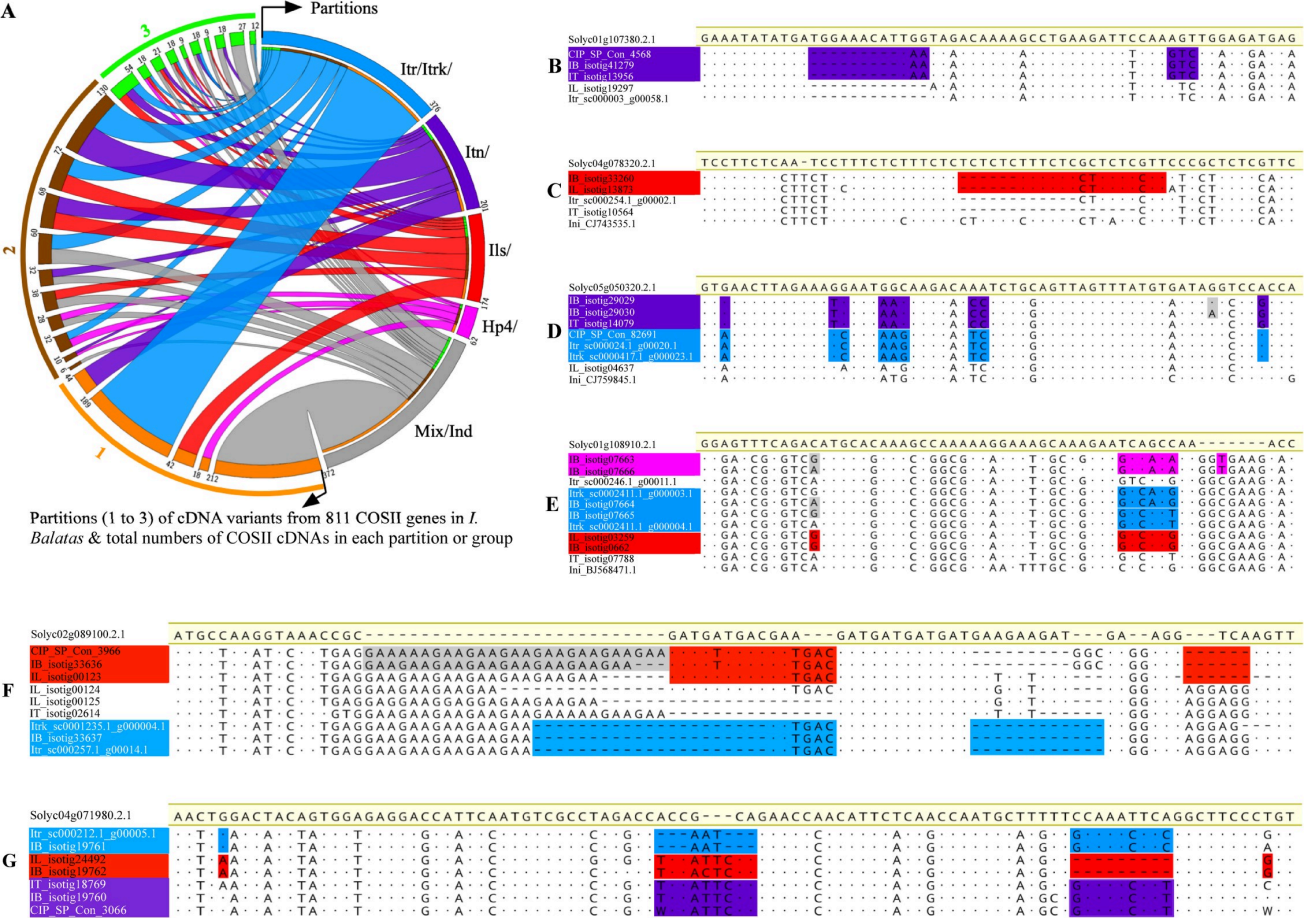
Phylogenetic partitions of the cDNA variants of 811 COSII genes in sweetpotato. Alignments of equal-consensus-length homologous regions from cDNA variants of 811 COSII genes from two sweetpotato clones (IB_ and CIP_ for those isotigs from the SC1149-19 and Tanzania) and from homologous COSII cDNAs from two *I*. *trifida* lines (Itr_ and Itrk_), one *I*. *littoralis* (IL_) and one *I*. *tenuissima* (IT_) accessions, one *I*. *nil* line (Inil_), and tomato (Solyc_) were used for the phylogenetic partitions of cDNA variants of the sweetpotato COSII genes. The Circos plot (**A**) summarized numbers and types of partitions or group that could be distinguished among cDNA variants of the COSII genes in sweetpotato, and total numbers of different COSII cDNAs that were identified in each of the four partitions and the Mix/Ind group. In 505 of the 811 alignments, single or multiple cDNA variants of a COSII gene from the two sweetpotato clones could be distinguished into only one (the orange-colored arcs) of the four variation partitions, the Itr/Itrk/(blue-colored), Itn/ (purple-colored) or Ils/(red-colored) partitions by their differentially conserved non-random clusters of interspecific variations with the corresponding reference homologs from the two *I*. *trifida* lines, *I*. *tenuissima* and *I*. *littoralis* accessions, respectively, and the Hp4/ (pink-colored) by their unshared non-random clusters of variations at the interspecific variable sites; or were placed into the Mix/Ind group (gray-colored) as their variations at the interspecific variable sites were of mixed (Mix/) or indistinguishable (Ind/) relatedness with those in the corresponding three reference homologs. In the rest of the 811 alignments, the cDNA variants of a COSII gene from the two sweetpotato clones were of two (brown-colored arcs) or three (green-colored arcs) types of interspecific variations, which could be differentiated into various combinations of partitions and the Mix/Ind group. The sub-alignments shown in **B** to **G** were two examples each for one, two and three partitions of cDNA variants of different sweetpotato COSII genes. In the alignments **D**, **E** and **F**, the gray-shaded variations between the two COSII cDNA variants from the same or different sweetpotato clones exemplified the intraspecific variations (alleles) between cDNA variants of the COSII gene in the same partition.

First, these local intragenic variations could be usually distinguished into two contrasting types. A more predominant type of variations, including SNPs, MNPs (Multinucleotide Polymorphisms) and Indels, among cDNA variants of many sweetpotato COSII genes were at the sites (e.g. those labeled by green-triangles in Fig 2A-2, 2A-3 and 2B) that were interspecifically variable among respective COSII cDNA homologs from sweetpotato and its three closest relatives, and were either differentially conserved in the reference homolog(s) from one or two of the three relatives (Fig 2A-3) or unique to the COSII cDNA variant(s) from sweetpotato (more details below). These differentially interspecifically conserved variations in cDNA variants of a sweetpotato COSII gene most likely were of distinct origins before speciation of sweetpotato, and were thus homoeoalleles among homoeologous loci of the COSII gene. Some additional variations (e.g. those labeled by maroon triangles in Fig 2A-2 and 2B) among cDNA variants of these sweetpotato COSII genes were not shared interspecifically in any of the reference cDNA homologs, and not at interspecifically variable sites. These intraspecific intragenic variations in a sweetpotato COSII cDNA variant were thus most likely alleles at one of the three homoeologous loci. Furthermore, some variations at interspecifically variable sites or other sites in cDNA variants of less than 5% of the COSII genes were not conserved in the same reference homolog as their neighboring interspecific variations, but shared in a second reference homolog(s), which suggested that they should be most likely derived from parallel mutations from the original residues as those at the same sites in the first reference homolog(s) before or after speciation of sweetpotato. These variations could not be easily determined to be homoeoalleles or alleles as they can be either type or both, but did not impair phylogenetic partition of the cDNA variants. An example of such variations can be seen in Fig 2A-3. The fifth residue “C” of the IB_isotig45341 was most likely a mutation from “A” as judged by the flanking differential variations homologous with those in IT_isotig18823 from *I*. *tenuissima*, and thus homoplasious to the “C” at the interspecifically variable site in the cDNA Itrk_sc0007727.1_g000001.1 from one of the two *I*. *trifida* lines. Thus, this SNP might be homoeoallelic to those in IB_isotig47343 and CIP_SP_Con_17460 from the two sweetpotato clones, and conceivably allelic to an unidentified one having the same “A” residue at the site as that in the IT_isotig18823.

Secondly, the interspecifically variable sites among cDNA homologs of a COSII gene from sweetpotato and its three closest relatives were present in statistically non-random clusters as determined by the Stephen’s statistical phylogenetic partition. These non-random clusters of variations among the COSII cDNA homologs in the alignments were usually spaced by nearly identical sequences of several tens to hundreds of base pairs for all the cDNA homologs from sweetpotato and its three closest relatives, as can be visually noticed in (Fig 2A-2, 2A-3 and 2B).

### Four differentiable variation partitions of sweetpotato COSII genes

By using the Stephen’s phylogenetic partition, cDNA variants of various sweetpotato COSII genes in the 811 alignments could be distinguished into three variation partitions, marked as the Itr/Itrk/, Itn/ and Ils/, based on their differential conservation of non-random clusters of interspecific variations with the corresponding reference homologs from *I*. *trifida*, *I*. *tenuissima* and *I*. *littoralis*, respectively, and into an additional Hp4/ partition by their sweetpotato-specific non-random clusters of interspecific variations. Additionally, one or multiple sweetpotato COSII cDNA variants (counted as a single different one) in one third of the alignments were of multiple mixed partition-types (Mix) of interspecific variations in different regions, implying homoeologous recombinations or chimeric assembly, or could not be clearly partitioned due to insufficient differential clusters of interspecific variations (Ind), indicating lack of phylogenetic differentiation. A total of 372 different COSII cDNAs of such types were included in the Mix/Ind group (Fig 3A). Please also note that since minor allelic variations between two sweetpotato COS II cDNA variants of the same interspecific variation partition are of no direct relevance to the phylogenetic study on the origin of their residing subgenome, the two COSII cDNAs were counted as one homoeoallele type in the summary Circos graph in Fig 3A.

**Fig 4:**
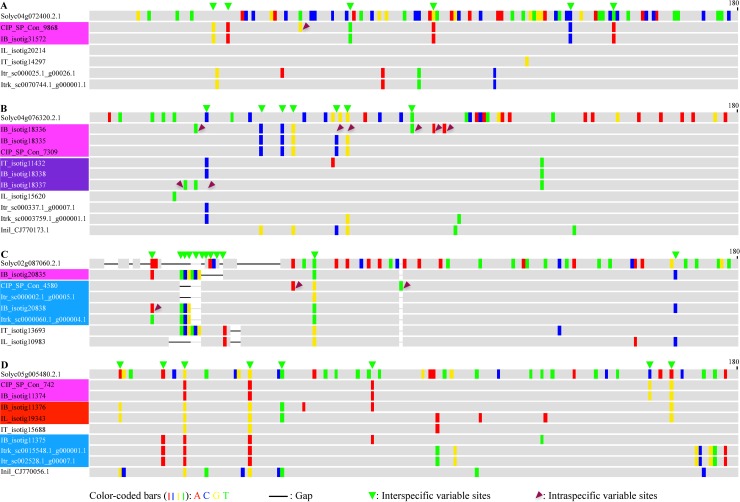
Illustration of the Hp4/ partition of cDNA variants from four sweetpotato COSII genes. In the zoom-out variation-highlighting views of four sub-alignments, an Hp4/ partition of the cDNA variant(s) from one or both of the two sweetpotato lines, which displayed distinct clusters of variations at interspecific variable sites as compared to those in all the homologs from the other four *Ipomoea* species and tomato, could be the only identifiable partition of cDNAs for a COSII gene (**A**), one of the two partitions of cDNA variants in combination with the Itn/ (**B**) or Itr/Itrk/ partition (**C**) for two COSII genes, and one of the three partitions of cDNA variants in combination with the Ils/ and Itr/Itrk/ partition for another COSII gene (**D)**. All the prefixes marking the species source of cDNAs and color-coding of the partitions were the same as in previous figures.

As summarized in the Circos graph (Fig 3A), among the 811 sweetpotato COSII genes, a majority of them (505) were of identifiable cDNA(s) of a single variation type in one of the four partitions or the Mix/Ind group. This indicated that a large percentage of the 505 COSII genes in sweetpotato, excluding some with unidentified variants due to coverage and analyses errors (e.g. out of variable regions), may not be differentiated at their respective homoeologous loci, but were of different lineages. The other two groups of 238 and 68 sweetpotato COSII genes were of two or three identifiable types of cDNA variants in different double or triple combinations of variation partitions or the Mix/Ind group, which demonstrated that at least ~38% of the 811 sampled COSII genes in sweetpotato should be partly or completely differentiated in two or three lineages among their respective three homoeolog triplets. Examples of single, double and triple identifiable variants of various COSII genes that could be differentiated into one, two and three partitions were illustrated with colored-block highlighting in Figs 3B–3G and 4A–4D. Please note that various gene tree methods were attempted, but usually failed to yield consistent results on the relatedness of partial cDNA homologs of a COSII gene form the five *Ipomoea* species in these alignments, indicating lack of robustness of the tree methods in dealing with the small-scale local variations in the relatively short (<1 kbp) sequence alignments.

### The largest Itr/Itrk/ partition

The Itr/Itrk/ partition included 376 different sweetpotato COSII cDNAs from single-partition ones of 189 COSII genes, and one of two or three cDNA variants of 147 and 40 COSII genes, respectively (Fig 3A). This disproportionally larger number of COSII cDNAs in the Itr/Itrk/ than those in each of the other three partitions implied that the expressed homoeologs from two or all three of the homoeolog triplets of some of these COSII genes should be undifferentiable so as to be all included in the same Itr/Itrk/ partition. Examples of cDNA variants of sweetpotato COSII genes that can be differentiated into double combinations of Itr/Itrk/ with Itn/, Ils/ or Hp4/ partitions, and triple combination of Itr/Itrk/ with Itn/ and Ils/, with Ils/ and Hp4/, were illustrated in Figs 3D, 3F and 4C and Figs 3G, 3E and 4D, respectively. More than half of the reference COSII cDNA homologs from the two *I*. *trifida* lines displayed partly different sets of allelic variations (Itr/ and Itrk/) at clustered interspecific variable sites, indicating two distinct genetic lineages, one or both of which may be shared in one or two cDNA variants of a sweetpotato COSII gene as illustrated with the blue highlighting in Figs 3E and 4C.

#### A distinct Itn/ partition

Although the reference COSII cDNAs from *I*. *tenuissima* were usually very close to and occasionally almost indistinguishable from corresponding cDNA homologs from *I*. *trifida*, they in most cases were of multiple differential variations at clustered interspecific variable sites to clearly distinguish a distinct phylogenetic variation partition of one or two cDNA variant(s) among those of a sweetpotato COSII gene, often in a clear contrast to the Itr/Itrk/ partition of another cDNA variant from the same gene, as those illustrated in Figs 2A-3 and 3B, 3D and 3G. Therefore, the Itn/ partition clearly differentiated a distinct tenuissima-like lineage of COSII genes in sweetpotato. This second largest Itn/ partition included 201 different sweetpotato COSII cDNAs from single-partition ones of 44 COSII genes, and one of the two or three cDNA variants of 120 and 37 COSII genes, respectively.

#### The Ils/ partition specified by COSII cDNA homologs in one of two gene lineages in *I*. *littoralis*

Two or more cDNA variants of less than 5% of the COSII genes from the same *I*. *littoralis* line displayed two contrasting types of variations at clustered interspecific variable sites, one including many littoralis-specific (relative to the other two reference species) variations (e.g. the IL_isotig00123 in Fig 3F and the IL_isotig07652 in Supplemental S1 Fig) and the other that were well conserved with those in the Itr/Itrk/ or the Itn/ reference cDNA homolog (e.g. the IL_isotig07653 in Supplemental S1 Fig and the IL_isotig00125 in Fig 3F). In addition, many different COSII cDNAs of single variation type from *I*. *littoralis* displayed either one of the two types of clustered interspecific variations. These two interspecific variation types between COSII cDNA variants or among different COSII cDNAs in *I*. *littoralis* differentiated two distinct COSII gene lineages, i.e. a littoralis-specific and a trifida-tenuissima-like one, which is more in line with them being expressed from paleo-homoeologous loci in a reduced paleotetraploid genome than from two paralogous loci in a regular diploid genome. Those *I*. *littoralis* COSII cDNAs displaying littoralis-specific variations at interspecific-variable sites were used as references to distinguish 174 sweetpotato COSII cDNAs into the Ils/, including 42 different single-partition COSII cDNAs, and one of the two or three cDNA variants in various double or triple partition combinations from 70 and 62 COSII genes, respectively. Examples of cDNA variants of various COSII genes that can be differentiated into a single Ils/, double combinations of Ils/ with Itr/Itrk/, and triple combination of Ils/ with Itr/Itrk/ and Hp4/ or with Itr/Itrk/ and Itn/ were illustrated with the red highlighting in Figs 3C, 3F, 3E and 4D and 3G, respectively. In a small number of alignments, the lack of a littoralis-specific Ils/ reference cDNA may have caused classification of certain sweetpotato COSSII cDNA variants in the Mix/Ind group.

#### The sweetpotato-specific Hp4/ partition

The sweetpotato COSII cDNA variants in the Hp4/ partition were distinguished by their unique clustered variations at interspecific variable sites, most of which were not traceable in any corresponding cDNA homologs from all the other four *Ipomoea* species in the alignments. This sweetpotato-specific Hp4/ partition had 62 different COSII cDNAs, including 18 single-partition ones, and one of the two or three cDNA variants in various double or triple partition combinations from 35 and 9 COSII genes. Examples of cDNA variants of sweetpotato COSII genes that could be differentiated into a single Hp4/, double and triple combinations of the Hp4/ with IT/ or Itr/Itrk/, and the Hp4/ with Itr/Itrk/ and Ils/ were illustrated with the pink highlighting in Figs 4A, 4B, 4C, 4D and 3E, respectively. This sweetpotato-specific Hp4/ variation partition of COSII cDNAs differentiated an additional lineage of COSII genes in sweetpotato.

### Partly and completely differentiated homoeologous COSII-marker regions of COSII genes

To further confirm differentiation (partly or completely) of homoeolog triplets of many COSII genes, we sought to sequence and compare genomic amplicon variants of the COSII-marker regions from two sweetpotato clones (SC1149-19 and LSP breeding line) and *I*. *tenuissima*. Since the original tomato COSII-marker primers anchored in exons were designed to amplify short introns of high allelic heterogeneity, the corresponding homologous COSII-marker regions in sweetpotato should likewise include more variable introns of homoeologs of various COSII genes, which has been sample-confirmed in our subsequent effort in developing genotype-by-sequencing markers using the sweetpotato COSII-marker amplicons (data not shown). Using 864 pairs of modified COSII-marker primers, we obtained 729 sets of genomic amplicon variants of COSII-maker regions of 729 out of the 811 COSII genes from the two sweetpotato clones and their homologs from *I*. *tenuissima*. Using both haplotype tree and phylogenetic partition methods, analyses of variations among COSII-marker amplicon variants of the 729 COSII genes from the two sweetpotato clones with the homologous partial cDNAs and COSII-marker genomic amplicons of corresponding COSII genes from *I*. *tenuissima* as references in alignments revealed four different levels of differentiation of the COSII-maker amplicon variants from these sweetpotato COSII genes.

**Fig 5:**
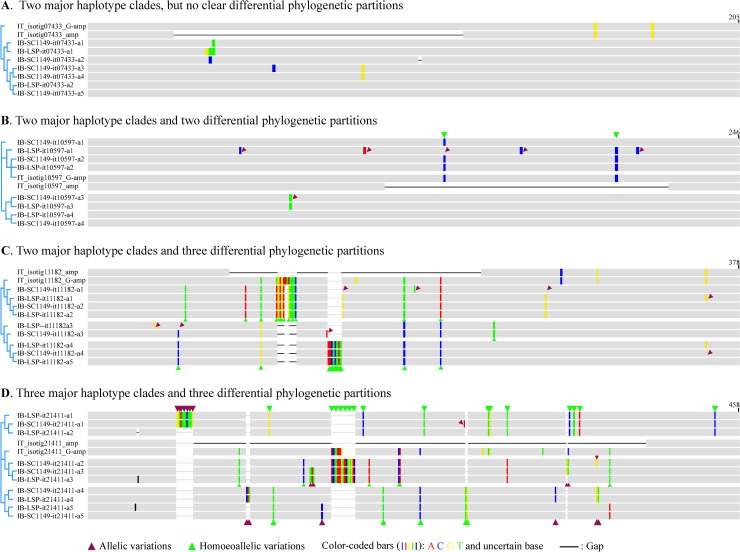
Illustration of four levels of variation differentiation of COSII-marker genomic amplicon variants among 729 sweetpotato COSII genes. The panels show variation-highlighting views of alignments of, and haplotype trees for the four sets of COSII-marker amplicon variants (_a1 to _a5) from four COSII genes in two sweetpotato clones (IB-SC1149- and IB-LSP-), as compared to their respective reference cDNA (_amp) and genomic amplicons (_G-amp) from corresponding homologous COSII-marker regions from *I*. *tenuissima* (IT_). The color-coded highlighting bars represent sequence variation(s) against consensus at the positions in each alignment. The black lines (gaps) connecting two horizontal gray bars for representing reference cDNA amplicons in each alignment delineate introns in respective COSII-marker genomic amplicons. In the panel B, C and D, distinct cluster(s) of variations that were exclusively shared by some amplicon variants in each set, and those that were shared with or different from those in the reference COSII-marker amplicons, distinguished the three sets of COSII-marker amplicon variants into two or three phylogenetic variation partitions, which are separated by wider gaps in the alignments. Those variations between partitions of amplicon variants were most likely homoeoalleles, and labeled by green triangles. Additional variations between the amplicons variants in the same partition were most likely true alleles, and were marked by brown triangles.

For 170 COSII genes, their COSII-marker amplicon variants displayed so small number of intragenic variations that no or two dubious major clades could be distinguished by corresponding haplotype trees, but without clear phylogenetic partition as illustrated in Fig 5A. For another 140 COSII genes, their COSII-marker amplicon variants could be clearly distinguished into two major clades on the haplotype trees, and correspondingly the tenuissima-like and -unlike partitions by shared or different clusters of interspecific variations between the amplicon variants and the reference homolog, as illustrated in Fig 5B. For the rest of 419 COSII genes, their COSII-marker amplicon variants could be either distinguished into two major clades, one of which, usually the tenuissima-unlike one, could be further distinguished into two partitions by additional interspecific variations shared by a subgroup of the amplicon variants as in Fig 5C, or three major clades corresponding to three interspecific variation partitions as in Fig 5D. Thus, the number of COSII genes that were partly or completely differentiated at homoeologous loci accounted for about 78% of the 729 sampled COSII gene in sweetpotato. The consistency of the variant grouping by the haplotype tree method and the phylogenetic partition with a higher resolution for the latter, as demonstrated in Fig 5B to 5D, also validate the phylogenetic partition method for grouping COSII cDNA variants. In addition, the intraspecific (brown-triangle labeled) variations and the clustered interspecific (green-triangle labeled) ones, as illustrated in Fig 5, could be also clearly distinguished in introns of at least one of COSII-marker amplicon variants from 559 sweetpotato COSII genes.

What is noteworthy in the above comparative analyses of cDNA and genomic amplicon variants of COSII genes is the predominance of interspecific (i.e. homoeoallelic) over intraspecific (i.e. allelic) variations among the partly and completely differentiated cDNA and genomic amplicon variants of more than two third of sampled COSII genes. This strongly argued against the frequently claimed high heterozygosity in sweetpotato in literature, and is more consistent with a very low heterozygosity due to very limited hybridizations between the vegetatively propagated genotypes due to widespread incompatibility and flowering unsynchronization among various sweetpotato genotypes, as compared to other crops. The observed genetic heterogeneity in sweetpotato should be mostly homoeoallelic, rather than allelic.

### Tracing ancestries of the four COSII genes lineages

**Fig 6:**
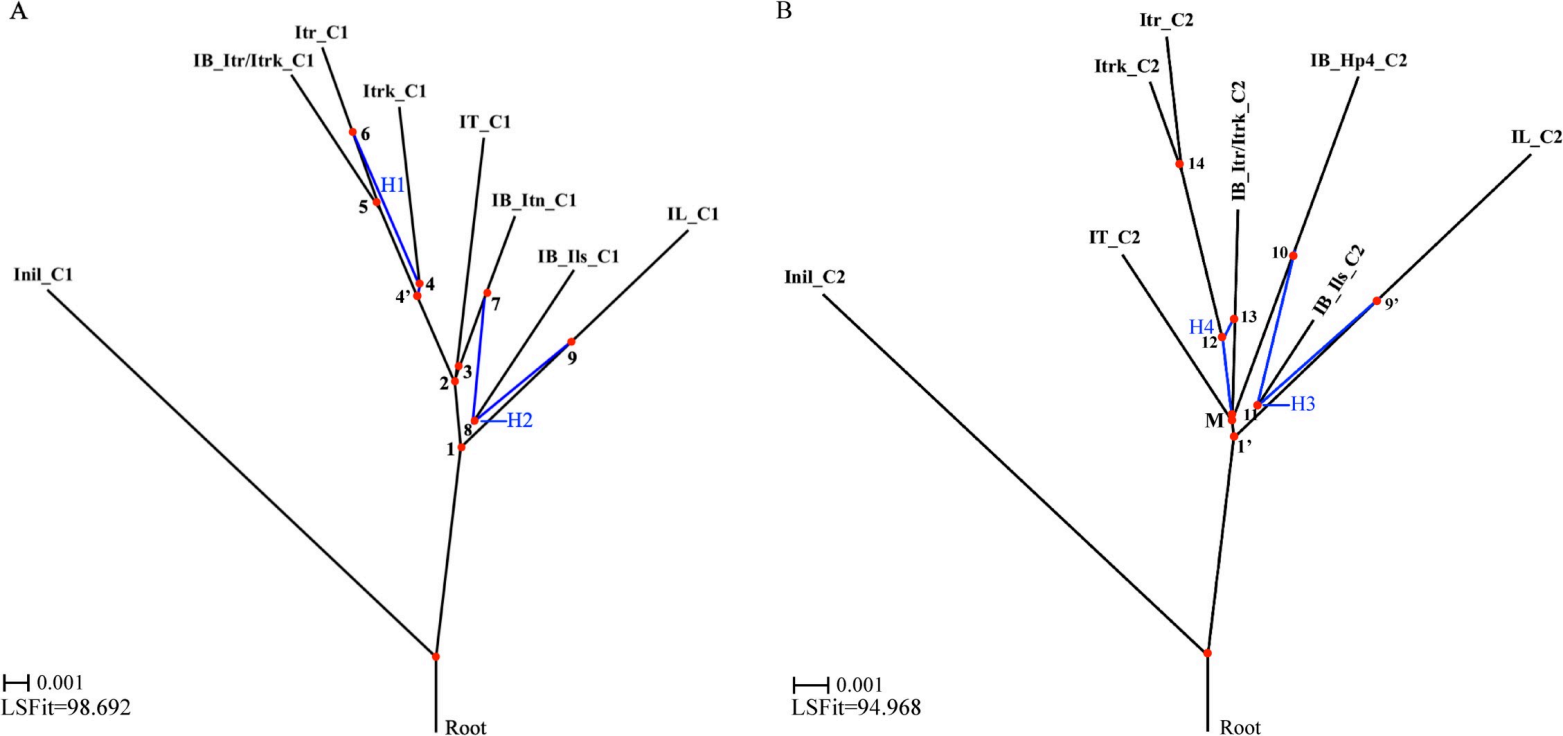
Two hybridization networks constructed from two concatenations of 16 (A) and 4 (B) individual alignments of partial cDNA homologs of 16 and 4 COSII genes from five *Ipomoea* species. The first concatenation (_C1) comprised three taxa (IB_Itr/Itrk_C1, IB_Itn_C1 and IB_Ils_C1) corresponding to 16 expressed COSII homoeolog triplets in the Itr/Itrk/, Itn/ and Ils/ partitions, and five taxa of corresponding COSII cDNA homologs from the two *I*. *trifida* lines (Itr_C1 and Itrk_C1), *I*. *tenuissima* (It_C1), *I*. *littoralis* (IL_C1), and *I*. *nil* (Inil_C1 as an outgroup). The second concatenation comprised three taxa (IB_Itr/Itrk_C2, IB_Hp4_C2 and IB_Ils_C2) corresponding to 4 expressed COSII homoeolog triplets in the Itr/Itrk/, Hp4/ and Ils/ partitions, and 5 taxa of corresponding COSII cDNA homologs from the two *I*. *trifida* lines (Itr_C2 and Itrk_C2), *I*. *tenuissima* (IT_C2) *I*. *littoralis* (IL_C2) and *I*. *nil* (Inil_C2, as an outgroup). The two hybridization networks were the HybridizationNetwork transformations of two split networks that were resolved from the LogDet distances between the 8 taxa each in the two concatenated alignment matrices by the SplitDecomposition method. The LSFit was the least squares fit between the pairwise distances of splits in the two hybridization networks and the taxa in the alignment matrices. The internal nodes that explicitly predict ancestors in the two networks were highlighted with red dots, and number labeled except the earliest one in the networks. The blue-colored splits indicated four detected hybridization events (H1 to H4).

To distinguish ancestries of the four differentiated lineages of COSII genes in sweetpotato, we constructed hybridization networks from the two above-mentioned alignment concatenations (variation highlighting view in Fig 2, and sequences in Supplemental S2 and S3 Figs). The two concatenations included two sets of fully differentiated expressed homoeolog triplets in two triple-partition combinations (Itr/Itrk/, Itn/ and Ils/, and Itr/Itrk/, Ils/ and Hp4/) from16 and 4 sweetpotato COSII genes, which were aligned with corresponding reference homologs from the three closes-relative species and one outgroup homolog from *I*. *nil*. Please note that these 16 and 4 COSII genes out of the 811 analyzed ones were all that could be identified to have three fully differentiated expressed homoeolog triplets in matching triple-partition combinations, and corresponding reference homologs of equal-consensus-length from all the other four *Ipomoea* species for proper alignment concatenations. The two hybridization networks (Fig 6) were surprisingly simple, and much more “tree” like. They are robust, albeit a much lower resolution in the second network, as phylogenies of the five taxa of the COSII cDNA homologs from *I*. *trifida*, *I*. *tenuissima*, *I*. *littoralis* and *I*. *nil* each in the two networks are consistent between the two networks, and more importantly with the taxonomy of the four species.

As the hybridization network is explicit in predicting ancestors [22], the nodes (red-dots) on the two network trees predict ancestors of the COSII multi-gene lineages in the *Ipomoea* species. The two hybridization networks revealed that two direct branching and one hybridization event (H2 or H3) could account for phylogenies of the set of expressed COSII homoeolog triplets of the particular triple-partition combination from sweetpotato in each network. First, the IB_Itn_C1 and the IB_Hp4_C2 taxa from sweetpotato branched in parallel, relative to all the taxa from three reference species and to the two matching hybrid nodes ancestral to the two taxa of the same Ils/ partition from sweetpotato, in the two networks. The IB_Itn_C1 taxon, representing the COSII homoeologs of a tenuissima-like lineage in sweetpotato, and the IT-C1 taxon from *I*. *tenuissima*, were sisters branched from the node N3, which was in turn a sister node of the N4’ leading to the Itrk_C1 and Itr_C1 taxa representing two genetic lineages in *I*. *trifida*. Thus, the IB_Itn_C1 taxon from sweetpotato should be an evolutionary cousin to the Itr_C1 and Itrk_C1 taxa from *I*. *trifida*, and probably originated from an ancestral species in a sibling relationship to *I*. *trifida*. Moreover, the IB_Hp4_C2 taxon, representing the COSII homoeologs of the sweetpotato-specific lineage in sweetpotato, descended from the incompletely resolved node M, which was also a progenitor to the other four taxa from three *Ipomoea* species. The parallel phylogenies of the IB_Itn_C1 and the IB_Hp4_C2 taxa in the two networks predicted that the two taxa from sweetpotato probably descended from one progenitor species hosting the N3 node. Secondly, the IB_Itr/Itrk_C1 and the IB_Itr/Itrk_C2 taxa representing the COSII homoeologs of trifida-like genetic lineages in sweetpotato also branched in parallel from the nodes N5 and M leading to the Itr_C1, Itr_C2 and Itrk_C2 taxa from *I*. *trifida* in the two networks. The IB_Itr/Itrk_C1 taxon from sweetpotato branched from the Itr_C1 lineage even more recently than the other genetic lineage, Itrk_C1, in *I*. *trifida* in the first network, and thus was probably derived directly from an *I*. *trifida* genotype. However, the IB_Itr/Itrk_C2 taxon from sweetpotato branched from a distant common ancestral node M to the Itr_C2 and Itrk_C2 taxa from *I*. *trifida*, and the IB_Hp4_C2 taxon from sweetpotato, displaying a deep coalescence pattern. The IB_Itr/Itrk_C2 taxon may thus represent those sweetpotato COSSII homoeologs that may have retained ancient variations of the corresponding homologs in a distant conman ancestral species to *I*. *trifida* and to the one that contributed the IB_Hp4_C2 taxon in sweetpotato due to incomplete lineage sorting (ILS) so that the taxon could still have been contributed from the same *I*. *trifida* genotype as the IB_Itr/Itrk_C1 taxon. Lastly, the IB_Ils_C1 and IB_Ils_C2 taxa, representing the COSII homoeologs of the littoralis-like genetic lineages in sweetpotato, were descendants of hybrid nodes N8 and N11 between an ancestor (N7 or N10) leading to the IB_Itn_C1 or IB_Hp4_C2 taxon and an ancestor (N9 or N9’) lineal to the IL_C1 or IL_C2 from *I*. *littoralis*, respectively. The two hybrid parents N9 and N9’that branched in parallel from the two most distant ancestral nodes (N1 and N1’) leading to all the other 6 taxa in the two networks were most likely hosted in one ancestral species. The other two hybrid parents N7 and N10 were also in parallel in the two networks, and would be causally in a second ancestral species. However, both pairs of hybrid parents were also direct ancestors to two additional interspecific taxa (IB_Itn_C1 and IL_C1, IB_Hp4_C2 and IL_C2) each in the two networks. This topology implied that the two parallel hybridizations in the two networks should be originated only from a hybridization of two autotetraploids in which the two pairs of hybrid parental nodes were duplicated. Additionally, the hybrid node N4 leading to the Itrk_C1 in the first network should be derived from an intraspecific introgression event, so as the N12 lineal to the Itr_C2 and Itrk_C2 in the second network if the IB_Itr/Itrk_C2 was contributed from an *I*. *trifida* genotype.

### A species tree embodying the two hybridization networks

**Fig 7:**
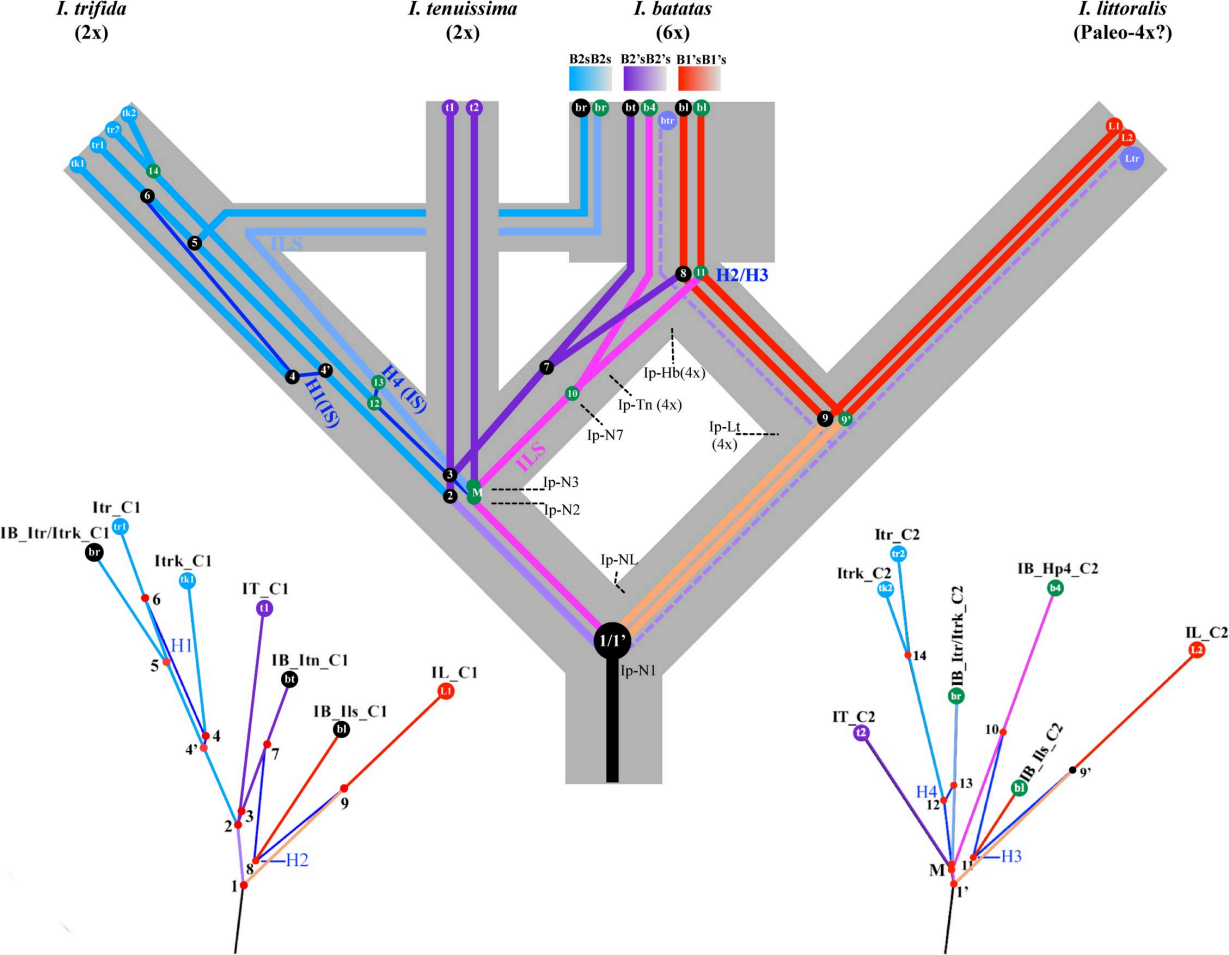
The most-parsimonious species tree embodying the two hybridization networks. The gray-shaded outline represents the most parsimonious species tree that could be reconciled with the two hybridization networks. The two hybridization networks minus their outgroups were fitted in the gray-shaded species tree trunks, having matching partition-color-coded splits, numbered nodes and hybridization events (H1 to H4 in blue) as those in the two redrawn color-coded hybridization networks minus their outgroups at the bottom left and right for comparison. The three pairs of sweetpotato COSII lineages fitted in the species tree and their corresponding taxa in the two hybridization networks were labeled as br, bt, bl and b4 for trifida-like, tenuissima-like, littoralis-like and sweetpotato-specific COSII lineages, respectively, in black (first network) and green (second network) color circles. The reference COSII lineages in the three sweetpotato relatives and their corresponding taxa in the two hybridization networks were labeled as tr or tk, t, and L for those from the two *I*. *trifida* lines, *I*. *tenuissima*, and *I*. *littoralis*, respectively, with subscripted number 1 or 2 indicating the first and second network in respective partition-color circles. The tree sections predicting ancestral species (diploid Ip-N1, -N2, -N3 and -N7, tetraploid Ip-Tn, -Lt and -Hb) to sweetpotato were delimited by dashed lines. The H1 and H4 hybridizations were modeled as intraspecific introgression (IS) in *I*. *trifida*. The br (IB_Itr/Itrk_C2) and b4 (IB_Hp4_C2) lineages (light-blue and pink colored, respectively) in the second network were modeled as outcomes of incomplete lineage sorting (ILS) in the tree. The dotted light-purple lines mark an additional predicted COSII lineage (btr) in sweetpotato and the trifida and tenuissima-like one (Ltr) in *I*. *littoralis*, which would have descended from an ancestral linage branched at the node N1/1’. The three color-gradient blocks underneath the subgenome designations were used to emphasize that only parts of these diploid subgenomes were differentiated as represented by the differentiated multi-gene COSII lineages.

To reconstruct species phylogenies accounting for the ancestries of the four lineages of sweetpotato COSII genes, we attempted the most parsimonious inference of a species tree that could be reconciled with the two hybridization networks minus the outgroup splits. The species tree shown in Fig 7 was mainly delineated by the two parallel H2 and H3 hybridizations in the two networks, and by the homoeologous relationships between the IB_Itn_C1 and IB_Ils_C1, and between the IB_Hp4_C2 and IB_Ils_C2 taxa in sweetpotato. A hypothesis of requiring the fewest evolutionary steps to cause the two hybridizations in the two networks would be two synchronous hybridizations of the two pairs of COSII lineages of different ancestries during an interspecific hybridization between their host tetraploids. Furthermore, the homoeologous relationships between the two taxa of each pair that was coupled by a hybridization in each network implied that the closest ancestors to each pair of the taxa should be hosted in two diploid subgenomes of a hybrid tetraploid.

The species tree predicted that the hexaploid sweetpotato was originated from a hybridization between a *I*. *trifida* genotype carrying the two trifida-like COSII genetic lineages (br in black and green circles) and a tetraploid hybrid (Ip-Hb) hosting two interspecific (inter-subgenomic) pairs of COSII lineages, i.e. the tenuissima-like (bt) and sweetpotato-specific (b4), and the two littoralis-like hybrid (bl in blue and green circles) lineages. The inferred tetraploid hybrid Ip-Hb could have been derived from a hybridization between an autotetraploid (Ip-Tn), which was from doubling of an earlier ancestral diploid (Ip-N7) genome in a sister and a niece kinship to those of the extant *I*. *tenuissima* and *I*. *trifida*, respectively, and another tetraploid (Ip-Lt), which was from doubling of an earlier ancestral diploid genome (Ip-NL) in a grand-aunt and an aunt kinship to those of the same two *Ipomoea* species. In others words, *I*. *trifida* would be a direct progenitor to only one (B_s_1’B_s_1’) of the constituent diploid subgenomes in sweetpotato. The other two subgenomes in sweetpotato would have been derived from hybrids of two pairs of duplicated diploid genomes (in Ip-Tn and Ip-Lt) in a respective niece and aunt kinship to that of *I*. *trifida*. They would thus be extremely closely related to, but not the same origin as the first sweetpotato subgenome from *I*. *trifida*. Additionally, *I*. *tenuissima* and *I*. *trifida* would be in a niece and aunt kinship, derived from the predicted ancestor Ip-N2 hosting the network nodes N2 and M, and in a third and second cousin kinship to *I*. *littoralis*, respectively. By the parsimony principle, *I*. *littoralis* was modeled as a paleo-tetraploid derived from the predicted Ip-Lt tetraploid. Since the three diploid subgenomes in sweetpotato were only partly differentiated as represented by the three inter-subgenomic pairs of differentiable lineages of some COSII genes, they were thus noted using the three color-gradients blocks under the subgenome designations.

In the species tree, the sweetpotato-specific COSII lineage (b4) was fitted as one that retained some ancient polymorphisms from the most distant ancestor Ip-N1 (hosting network nodes 1/1’), i.e. resulting from an ILS, which was consistent with the fact that the expressed homoeologs of COSII genes in this lineage (Hp4/) carried unique ancient variations that could not be traced in homologs from the three closest relatives, nor in most cases from *I*. *nil*. Furthermore, based on the observed Itr-Itn-like second lineage (Ltr) for variants of some COSII genes in *I*. *littoralis*, the second subgenome in sweetpotato was predicted to also carry additional Itr-Itn-like btr lineages (dotted light-purple lines) in place of the Itn-like bt or the sweetpotato-specific b4 lineage for some COSII genes, which could have been carried over from the tree-predicted earlier ancestor (Ip_Lt) to *I*. *littoralis* during its hybridization with the predicted autotetraploid Ip-Tn. Just as expressed homoeologs of the Ltr lineage from some COSII genes in *I*. *littorali*s were almost indistinguishable from their homologs from *I*. *trifida* or *I*. *tenuissima*, the expressed homoeologs of the btr lineage from some sweetpotato COSII genes would have been too close to those of the trifida-like (Itr/Itrk/) or tenuissima-like lineage (Itn/) from the same COSII genes to be distinguished by the partition method, and thus hybridization not detectable. This predicted btr lineage of COSII genes in sweetpotato could have contributed to the disproportionally higher number of COSII cDNAs in the Itr/Itrk/ and Itn/ partitions, as these cDNAs could have been placed in the Itr/Itrk/ or Itn/ partition. The ancestral lineage to the predicted btr and the Ltr lineages was simplistically modeled as a distinct genetic lineage in an autotetraploid Ip_Lt and an earlier diploid Ip_NL ancestor due to lack of information. Additionally, the H1 and H4 hybridizations detected by the two networks were modeled on the species tree to be derived from intraspecific introgressions (IS) between genotypes of *I*. *trifida*, as explained in the network reconstruction.

## Discussion

The long-lasting disagreement on an auto- versus allohexaploid sweetpotato was rooted in two lines of phylogenetic signals or data that were not consistent with either one of the two mutually exclusive polyploidy types. There has to be an additional model that could account for both lines of phylogenetic signals in sweetpotato. Stebbins actually distinguished an intermediate polyploidy, the segmental allopolyploidy, in addition to the auto-and allopolyploidy [31]. Autopolyploids arise through duplication of a genome [31], or crossing between individuals of genetically distinct lineages within a species [32], and thus have identical or very similar subgenomes. Allopolyploids originate from interspecific hybridization concomitant with genome doubling [31, 32], and thus contain two or more distinct subgenomes. In contrast, segmental allopolyploids carry more than two partly differentiated genomes, display both bivalent and multivalent chromosome pairing [31, 33], and can be therefore very difficult to separate from true autopolyploids, especially if they were derived from hybridization of closely related species [34]. This may be exactly what best describe the sweetpotato hexaploidy based on our detailed comparative phylogenetic analyses of variants of sweetpotato COSII genes and their homologs in its closest relatives in a very large sample size, and two very early cytological studies on the meiotic chromosomal pairing in sweetpotato [3, 5].

First, the three diploid subgenomes of sweetpotato are very closely related, but not without phylogenetic differentiation. Our phylogenetic partition analyses of the 811 sets of aligned homologous cDNA regions from as many COSII genes in the five *Ipomoea* species, and combined haplotype-tree and phylogenetic partition analyses of genomic amplicon variants from COSII-marker regions of 729 out of the 811 COSII genes in sweetpotato and *I*. *tenuissima* revealed that variations between the cDNA variants of and between COSII-marker genomic amplicon variants of many sweetpotato COSII genes, and between COSII homologs from sweetpotato and its three closest relatives were mostly less than 5%, but displayed features and interspecifically conserved patterns of phylogenetic differentiation implication. 1. Two types of intragenic variations could be clearly distinguished in the cDNA and COSII-marker amplicon variants of many COSII genes (Figs 2, 3, 4 and 5). The predominant interspecific variations (homoeoalleles) were non-randomly clustered, interspecifically well conserved with one of the reference homologs from three closest relatives or sweetpotato-specific, whereas the minor intraspecific ones (true alleles) were randomly distributed mostly at non-interspecifically variable sites, and usually sweetpotato-specific. 2. Four species-homolog-specified partitions (Itr/Itrk/, Itn/, Ils/ and Hp4/) of the interspecific variations, and maximal three out of the four partitions in various combinations could be distinguished among different COSII cDNAs, and between variants of many COSII genes, respectively (Fig 3). 3. Either one or both of the tenuissima- or littoralis-specified partitions (Itn/ and Ils/) of interspecific variations were clearly differentiated from the trifida-specified one (Itr/Itrk/) among cDNA variants of many COSII genes. Furthermore, interspecific variations in some COSII cDNAs in the sweetpotato-specific partition (Hp4/) bore little resemblance to those in cDNA homologs from any of the three closest relatives and *I*. *nil*. These results did not support the conclusion of an autopolyploid origin of sweetpotato from *I*. *trifida* based on a closer relatedness of “alleles” (from haplotype calling) of 307 sampled single-copy regions in sweetpotato to each other and to those in *I*. *trifida* than to “alleles” in any other extant relatives in a recent study [17].

Secondly, the partial or complete phylogenetic differentiation between expressed homoeologs or homoeologs of a portion of sampled COSII genes in sweetpotato points to partly differentiated subgenomes. A total of 238 and 68 COSII genes out of the 811 sampled ones in sweetpotato were of two or three phylogenetically differentiated cDNA variants in various double or triple combinations of species-specified variation partitions or the Mix/Ind group (Fig 3A), demonstrating that partly or completely differentiated homoeolog triplets of the respective COSII genes. Furthermore, the combined phylogenetic partition and gene tree analyses of COSII-marker amplicon variants from 729 COSII genes revealed a higher percentage of COSII genes that are partly (140, or 21.7%) or completely (419, or 57.5%) differentiated at homoeologous loci. Thus, some of the 293 and 212 sweetpotato COSII genes that had one identifiable type of cDNA variants in either one of the four variation partitions and in the Mix/Ind group, respectively, may be differentiated, but one or two of their cDNA variants unidentified. Nonetheless, a substantial proportion of these 505 sweetpotato COSII genes should not be phylogenetically differentiated at their respective homoeologous loci. On the other hand, these COSII genes without apparent homoeoallele differentiation could have one or multiple cDNA variants in different species-defined variation partitions (Fig 3A), implying a probable presence of many different COSII families of identical or non-differentiable fraternal homoeolog triplets in the genome. This unusual pattern of vertical phylogenetic differentiation in lineages among various COSII genes in each diploid subgenome and horizontal non-differentiation among inter-subgenomic homoeolog triplets of COS II genes could be explained by homogenization of the homoeologous loci of these COS II genes to one of their originally differentiated homoeolog triplets in different variation partitions (lineages), possibly through homoeologous recombination [35], nonreciprocal translocations as in *Brassica napus* [36, 37] and inter-subgenomic chromosomal exchanges that are often associated with closely related subgenomes in polyploids [33], or by homoeolog triplets of extremely closely related, but different ancestries. Had it been without references of the three species-defined variation partitions to distinguish different lineages of these COSII genes of different single variation type in a subgenome, the non-differentiation of their expressed homoeologs could have misled one to conclude an autopolyploid sweetpotato.

It is worth mentioning that the three partly differentiated diploid subgenomes in sweetpotato had been unequivocally recognized in an overlooked earlier study on karyological characteristics and chromosomal pairing at pachytene in sweetpotato, which concluded that “i) the three parental genomes are partly homologous, (ii) two of the genomes show closer homology to one another than to the third and (iii) the three genomes differ with respect to one or more of the eight chromosomal types occurring singly” [5]. The results of our phylogenetic analyses of COSII genes in sweetpotato and the reconstructed phylogenies of three subgenomes on the inferred species tree (Fig 7) were consistent with the first and second conclusions of the study, respectively.

Thirdly, the four species-specified variation partitions of different COSII cDNAs distinguished four COSII gene lineages of three closely related, but distinct ancestries, which could have been brought together in sweetpotato only by hybridization of closely related progenitor species. The two hybridization networks (Fig 6) predicted that the taxa of the expressed homoeolog triplets in the Itr/Itrk/, Itn/ and Ils/, and in the Itr/Itrk/, Hp4/ and Ils/ partitions from the 16 and 4 sweetpotato COSII genes, respectively, differentiated three evolutionary lineages of different ancestries each among the homoeolog triplets of these COSII genes, i.e. the trifida-like lineages (Itr/Itrk/) of hybrid ancestries within *I*. *trifida*, the tenuissima-like (Itn/) or the sweetpotato-specific (Hp4/) lineages sharing apical ancestors with the COSII lineages in *I*. *tenuissima*, and the littoralis-like (Ils/) lineages with apical ancestors from hybridizations between two pairs of COSII lineages sharing ancestors with those in *I*. *tenuissima* and in a progenitor of *I*. *littoralis*. The substitution of the Itn/ by the Hp4/ partition for the taxon (IB_Hp4_C2) of the expressed homoeolog variants from the 4 sweetpotato COSII genes and the parallel topologies of the two matching splits leading to the two taxa (IB_Itn_C1 and IB_Hp4_C2) in the two networks indicated that the sweetpotato-specific (Hp4/) and the tenuissima-like lineages (Itn/) were probably different genetic lineages within one ancestral species. The inferred species tree (Fig 7) further predicted that the three evolutionary lineages of homoeolog triplets of these two sets (16 and 4) of COSII genes, so as their residing subgenomes in sweetpotato, most likely were of different species origins from *I*. *trifida* as an immediate progenitor and two of its close-relative species (tree-predicted Ip-Tn and Ip-Lt) as secondary ones, and that the sweetpotato-specific (Hp4/) lineage was most likely remnant of an ancient one from the N1/1’ due to ILS in the same ancestor (Ip-N3) as the tenuissima-like one.

Therefore, by the tree-predicted species parentage, sweetpotato should be an allohexaploid originated from hybridization of two closely related species and subsequent whole-genome duplication. However, the three diploid subgenomes of close-relative origins could have retained a high degree of similarity or been partly “homologous”, as concluded from the earlier cytological study [5], probably especially between some pairs or triplets of homoeologous chromosomes to allow multivalent pairing, consequently resulting in an inheritance resembling that of an autopolyploid. Such autopolyploid-like chromosome pairing has been well known in some allopolyploids including wheat [38]. Furthermore, frequent homoeologous recombinations could have facilitated homogenization of some homoeologous parts of the three subgenomes as indicated by those one-partition expressed homoeologs of many COSII genes, which may have made these parts of the subgenomes in sweetpotato even more resembling those of an autopolyploid. Thus, the sweetpotato hexaploid should not be a true type to either an allopolyploid, or to an autopolyploid, but best described by the concept of a segmental allohexaploid. Following the previous genome designation for a synthetic hexaploid hybrid resembling sweetpotato [9], B1B1/B2B2/B2B2, we proposed the use of B_s_1’ B_s_1’/ B_s_2’B_s_2’/ B_s_2B_s_2 genome designation, where the subscribed “S”, the prime symbol and the shared number “2” indicate “segmental” and “hybrid lineages”, and more similarity between the two subgenomes of the *I*. *trifida* and its niece species origins, respectively.

In this study, we demonstrated that the relatively small variations among homoeologs from the three subgenomes in sweetpotato, were of phylogenetic significance when analyzed using appropriate references and phylogenetic models, and could allow tracing phylogenies of their residing subgenomes. Our understanding on the origin and reorganization of the hexaploid sweetpotato genome is currently very limited. More in-depth phylogenetic studies on these issues are still much needed, and could employ the inferred species tree from this study as a testing model. While future molecular phylogenetic studies will certainly benefit by the rapidly growing NGS datasets from sweetpotato and related species in the *Ipomoea* section, extreme caution would have to be exercised to maximally reduce inclusion of inter-homoeolog assembly chimeras. These chimeras could mask and distort phylogenetic differentiation among homoeologs so as to generate misleading results. The NGS chimeric assembly problem is particularly severe in sweetpotato, at least partially due to the non-random clustering of small variations between homoeologs. Unfortunately, in our tests, no current major assembly algorithms or software packages could effectively deal with the problem. We resorted to cross-references between different assemblies of the same sets of reads, and between those from two different lines or varieties of the same species for maximal reduction of chimers in this study. This problem of assembly chimeras may prove to be a major deterrent or obstacle for the uses of NGS data from sweetpotato.

## Supporting information

S1 Fig Two distinct lineages of cDNA variants of a COSII gene in *I*. *littoralis*. One of the two cDNA variants of a COSII gene from *I*. *littoralis*, IL_isotig07652, differs from the other variant by a 25-bp indel (clustered interspecific variable sites) that was specific to *I*. *littoralis* relative to the other two reference species. The 25-bp indel was shared only by one of the homologous COSII cDNA variants, IB_isotig31171, from sweetpotato, and thus could partition the COSII variant into the Ils/ (red-shaded). The other cDNA variant of the *I*. *littoralis* COSII gene, IL_isotig07653, was more trifida-like, sharing variations at clustered interspecific variable sites with Itr_sc000187.1_g00033.1 from *I*. *trifida* (gray-shaded). The two cDNA variants of the COSII gene from *I*. *littoralis* thus seemed to be of two distinct evolution lineages, and better explained as ones derived from two homoeologous loci in a paleo-tetraploid, rather than from paralogs or as alleles at one locus in the extant *I*. *littoralis* line. The other cDNAs variants, IB_isotig31172, of the COSII gene from sweetpotato could be distinguished into the Itn/ (purple-colored).
(TIF)

S2 Fig Concatenated sequences of 16 alignment blocks of homologous cDNA regions from as many COSII genes in five *Ipomoea* species. The 16 alignment blocks are separated by columns of “-N (series)-” with the number of Ns in the series in front of each block marking its order in the concatenation. The concatenation contains three rows of corresponding cDNA variant triplets (IB_Itr/Itrk_C1, IB_Itn_C1 and IB_Ils_C1), which are in the Itr/Itrk/, Itn/ and Ils/ partitions, respectively, from the 16 sweetpotato COSSII genes, five rows of partition reference cDNA homologs from two *I*. *trifida* lines (Itr_C1 and Itrk_C1), *I*. *tenuissima* (It_C1) and *I*. *littoralis* (IL-C1), and the homolog from *I*. *nil* (Inil_C1) as an outgroup.
(PDF)

S3 Fig Concatenated sequences of 4 alignment blocks of homologous cDNA regions from as many COSII genes in five *Ipomoea* species. The 4 alignment blocks are separated by columns of “-N (series)-” with the number of “N”s in the series in front of each block marking its order in the concatenation. The concatenation contains three rows of corresponding cDNA variant triplets (IB_Itr/Itrk_C2, IB_Ils_C2 and IB_Hp4_C2), which are in the Itr/Itrk/, Ils/ and Hp4/ partitions, respectively, from the 4 COSSII genes in *I*. *batatas*, five rows of partition reference cDNA homologs from two *I*. *trifida* lines (Itr_C2 and Itrk_C2*)*, *I*. *tenuissima* (It_C2) and *I*. *littoralis* (IL-C2), and the cDNA homolog from *I*. *nil* (Inil_C2) as an outgroup.
(PDF)
