# Supplementary figures and images for: Hexaploid sweetpotato (Ipomoea batatas (L.) Lam.) may not be a true type to either auto- or allopolyploid
Source: PLoS One. 2020 Mar 3;15(3):e0229624. doi: 10.1371/journal.pone.0229624 (PMC7053752; doi:10.1371/journal.pone.0229624)

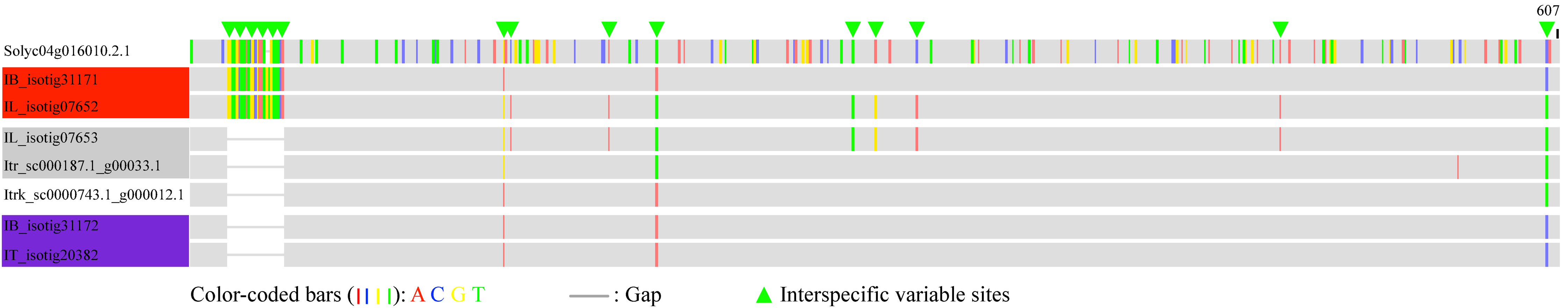

Supplement: S1 Fig — One of the two cDNA variants of a COSII gene from I. littoralis, IL_isotig07652, differs from the other variant by a 25-bp indel (clustered interspecific variable sites) that was specific to I. littoralis relative to the other two reference species. The 25-bp indel was shared only by one of the homologous COSII cDNA variants, IB_isotig31171, from sweetpotato, and thus could partition the COSII variant into the Ils/ (red-shaded). The other cDNA variant of the I. littoralis COSII gene, IL_isotig07653, was more trifida-like, sharing variations at clustered interspecific variable sites with Itr_sc000187.1_g00033.1 from I. trifida (gray-shaded). The two cDNA variants of the COSII gene from I. littoralis thus seemed to be of two distinct evolution lineages, and better explained as ones derived from two homoeologous loci in a paleo-tetraploid, rather than from paralogs or as alleles at one locus in the extant I. littoralis line. The other cDNAs variants, IB_isotig31172, of the COSII gene from sweetpotato could be distinguished into the Itn/ (purple-colored). (TIF) [file pone.0229624.s001.tif]
