# Supplementary material for: Hexaploid sweetpotato (Ipomoea batatas (L.) Lam.) may not be a true type to either auto- or allopolyploid
Source: PLoS One. 2020 Mar 3;15(3):e0229624. doi: 10.1371/journal.pone.0229624 (PMC7053752; doi:10.1371/journal.pone.0229624)
Supplement: S2 Fig — The 16 alignment blocks are separated by columns of “-N (series)-” with the number of Ns in the series in front of each block marking its order in the concatenation. The concatenation contains three rows of corresponding cDNA variant triplets (IB_Itr/Itrk_C1, IB_Itn_C1 and IB_Ils_C1), which are in the Itr/Itrk/, Itn/ and Ils/ partitions, respectively, from the 16 sweetpotato COSSII genes, five rows of partition reference cDNA homologs from two I. trifida lines (Itr_C1 and Itrk_C1), I. tenuissima (It_C1) and I. littoralis (IL-C1), and the homolog from I. nil (Inil_C1) as an outgroup. (PDF) [file pone.0229624.s002.pdf]

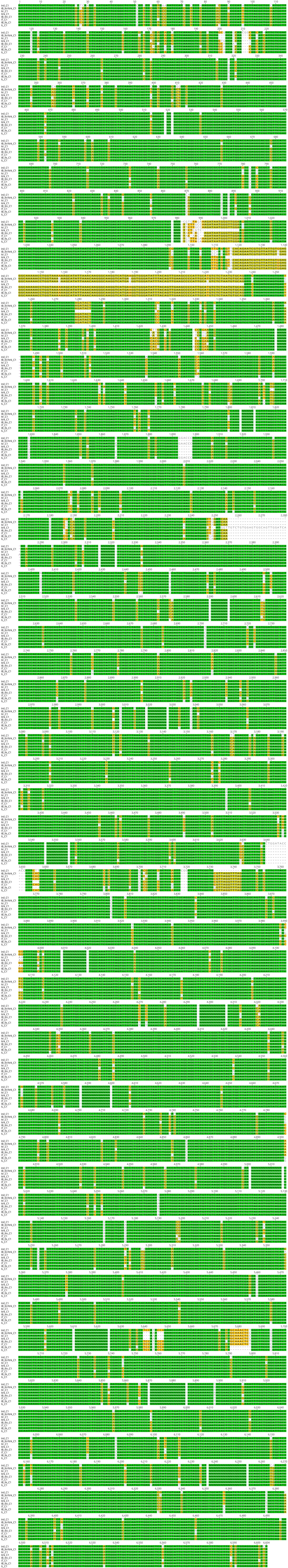

Identity of cDNAs in the concatenation 1

**Position No.**	**Solyc- reference homolog**	**IB_isotig identifer**	**Gene Identity**
**1**	Solyc03g117690.2.1	IB_isotig22259, -60 and -61	Aspartyl protease family protein At5g10770-like
**2**	Solyc06g066000.1.1	IB_isotig35725 and _26, and IB_isotig48839	Predicted ATP synthase submit b’
**3**	Solyc08g075850.2.1	IB_isotig45341and-43, and CIP_SP_Con_17460	50S ribosomal protein L24-like
**4**	Solyc08g077790.2.1	IB_isotig07848, -49 and -52)	Unknown
**5**	Solyc08g083260.2.1	IB_isotig22117, -18 and -19	Predicted snurportin-1
**6**	Solyc10g083870.1.1	IB_isotig27018, -19 and -20	Tankyrase-1-like
**7**	Solyc11g012840.1.1	IB_isotig21076, -77 and -78	BTB/POZ domain-containing protein At1g21780-like
**8**	Solyc09g090330.2.1	IB_isotig14185, -86 and -87	Harpin binding protein 1 (HrBP1)-like
**9**	Solyc02g084240.2.1	IB_isotig11992, -93, and -94	H1 histone-like
**10**	Solyc09g015820.2.1	IB_isotig25967, -68 and-69	Thioredoxin H9-lik
**11**	Solyc03g080100.2.1	IB_isotig22586, -87 and-88	Predicted heavy metal-associated isoprenylated plant protein 9
**12**	Solyc01g081190.2.1	IB_isotig18950 and -51, and CIP_SP_Con_1452	unknown
**13**	Solyc07g005390.2.1	IB_isotig19289 and-90, and CIP_SP_Con_5738	Non-phosphorylating glyceraldehyde dehydrogenase (GapN)-like
**14**	Solyc04g071980.2.1	IB_isotig19760, -61and -62	unknown
**15**	Solyc09g008920.2.1	IB_isotig19692, -93 and -94	heterodimeric geranylgeranyl pyrophosphate synthase small subunit-like
**16**	Solyc12g018990.1.1	IB_isotig22568, -69 and -70	Uridine nucleosidase 2-like
