# Supplementary material for: Hexaploid sweetpotato (Ipomoea batatas (L.) Lam.) may not be a true type to either auto- or allopolyploid
Source: PLoS One. 2020 Mar 3;15(3):e0229624. doi: 10.1371/journal.pone.0229624 (PMC7053752; doi:10.1371/journal.pone.0229624)
Supplement: S3 Fig — The 4 alignment blocks are separated by columns of “-N (series)-” with the number of “N”s in the series in front of each block marking its order in the concatenation. The concatenation contains three rows of corresponding cDNA variant triplets (IB_Itr/Itrk_C2, IB_Ils_C2 and IB_Hp4_C2), which are in the Itr/Itrk/, Ils/ and Hp4/ partitions, respectively, from the 4 COSSII genes in I. batatas, five rows of partition reference cDNA homologs from two I. trifida lines (Itr_C2 and Itrk_C2), I. tenuissima (It_C2) and I. littoralis (IL-C2), and the cDNA homolog from I. nil (Inil_C2) as an outgroup. (PDF) [file pone.0229624.s003.pdf]

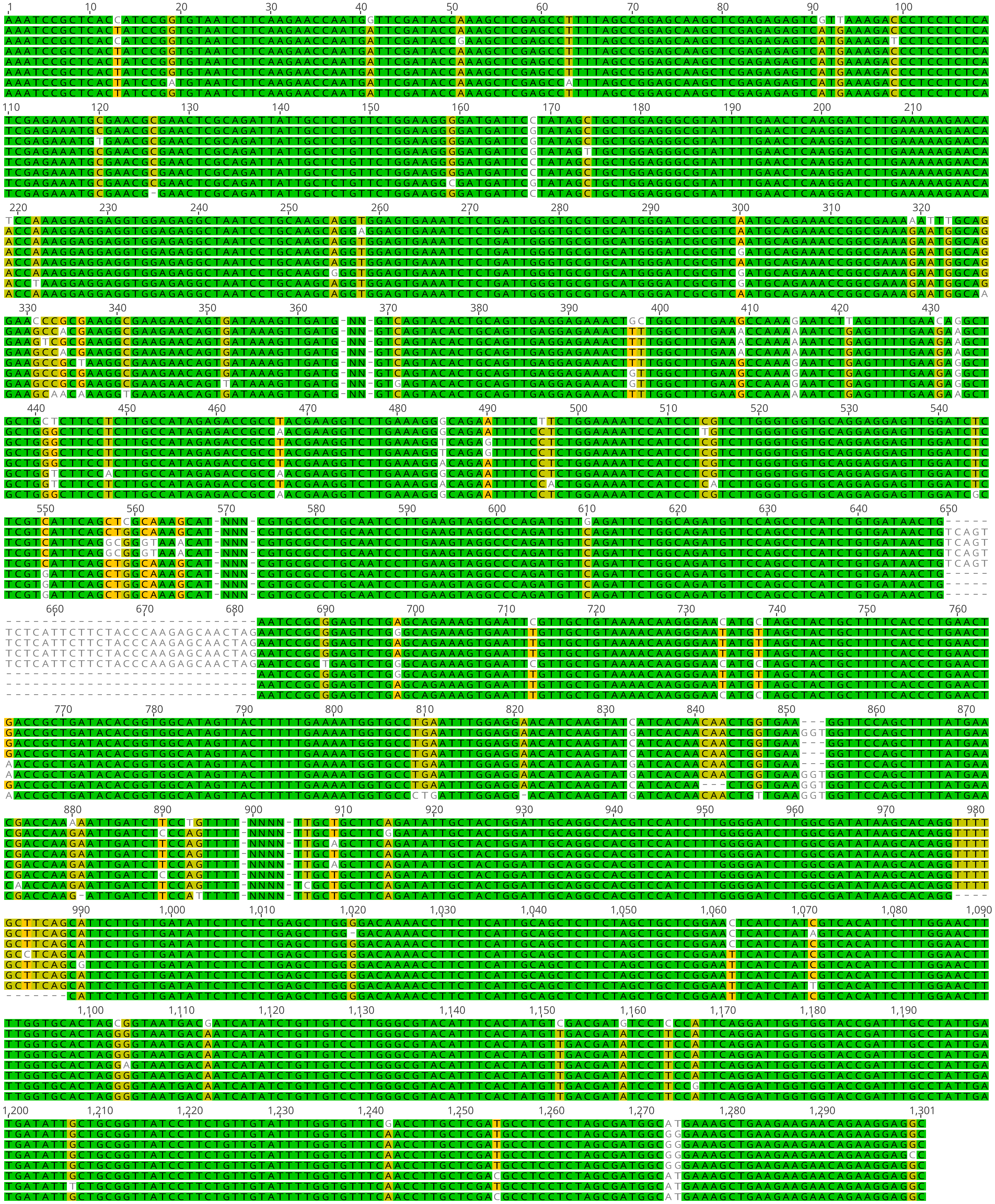

Identity of cDNAs in the concatenation 2

Position No.	Solyc- reference homolog	IB_isotig identifer	Gene Identity
**1**	Solyc01g108910.2.1	IB_isotig07664, -65 and -66	CCG-binding protein 1-like
**2**	Solyc05g005480.2.1	IB_isotig11374, -75, and -76	Predicted 2-methylene-furan-3-one reductase (EO)
**3**	Solyc11g069290.2.1	IB_isotig33727and-28, and CIP_SP_Con_24862	pyridoxine biosynthesis protein (Pdx2)-like
**4**	Solyc08g081770.2.1	IB_isotig18851, -52 and -54)	GDT1-like protein 1
